# Phylogenetic relationships, biofilm formation, motility, antibiotic resistance and extended virulence genotypes among *Escherichia coli* strains from women with community-onset primitive acute pyelonephritis

**DOI:** 10.1371/journal.pone.0196260

**Published:** 2018-05-14

**Authors:** Arianna Pompilio, Valentina Crocetta, Vincenzo Savini, Dezemona Petrelli, Marta Di Nicola, Silvia Bucco, Luigi Amoroso, Mario Bonomini, Giovanni Di Bonaventura

**Affiliations:** 1 Department of Medical, Oral, and Biotechnological Sciences, Laboratory of Clinical Microbiology, “G. d’Annunzio” University of Chieti-Pescara, Chieti, Italy; 2 Center of Excellence on Aging and Translational Medicine (CeSI-MeT), Laboratory of Clinical Microbiology, “G. d’Annunzio” University of Chieti-Pescara, Chieti, Italy; 3 "Spirito Santo" Hospital, Laboratory of Clinical Microbiology and Virology, Pescara, Italy; 4 School of Pharmacy, Microbiology Unit, University of Camerino, Camerino, Italy; 5 Department of Medicine, Nephrology and Dialysis Unit, “G. d'Annunzio” University of Chieti-Pescara, Chieti, Italy; Animal and Plant Health Agency, UNITED KINGDOM

## Abstract

The present work set out to search for a virulence repertoire distinctive for *Escherichia coli* causing primitive acute pyelonephritis (APN). To this end, the virulence potential of 18 *E*. *coli* APN strains was genotypically and phenotypically assessed, comparatively with 19 strains causing recurrent cystitis (RC), and 16 clinically not significant (control, CO) strains. Most of the strains belong to phylogenetic group B1 (69.8%; *p*<0.01), and APN strains showed unique features, which are the presence of phylogroup A, and the absence of phylogroup B2 and non-typeable strains. Overall, the most dominant virulence factor genes (VFGs) were *ecpA* and *fyuA* (92.4 and 86.7%, respectively; *p*<0.05), and the mean number of VFGs was significantly higher in uropathogenic strains. Particularly, *papAH* and *malX* were exclusive for uropathogenic strains. APN and RC strains showed a significantly higher prevalence of *fyuA*, *usp*, and *malX* than of CO strains. Compared to RC strains, APN ones showed a higher prevalence of *iha*, but a lower prevalence of *iroN*, *cnf1*, and *kpsMT-II*. Hierarchical cluster analysis showed a higher proportion of two gene clusters (*malX* and *usp*, and *fyuA* and *ecpA*) were detected in the APN and RC groups than in CO, whereas *iutA* and *iha* clusters were detected more frequently in APN strains. The motility level did not differ among the study-groups and phylogroups considered, although a higher proportion of swarming strains was observed in APN strains. Antibiotic-resistance rates were generally low except for ampicillin (37.7%), and were not associated with specific study- or phylogenetic groups. APN and RC strains produced more biofilm than CO strains. In APN strains, *iha* was associated with higher biofilm biomass formation, whereas *iroN* and *KpSMT-K1* were associated with a lower amount of biofilm biomass. Further work is needed to grasp the virulence and fitness mechanisms adopted by *E*. *coli* causing APN, and hence develop new therapeutic and prophylactic approaches.

## Introduction

Acute pyelonephritis (APN) is a very common community infection in women [1]. Although the associated mortality is only 0.7%, APN sometimes progresses to sepsis, uremia and multi-organ failure and, consequently, there can be dismal outcomes [2].

Pyelonephritis is usually caused by ascent of bacteria from the bladder. The most common pathogens in APN belong to the *Enterobacteriaceae* family, with *Escherichia coli* representing the leading cause in more than 80% of cases [3, 4]. Other microorganisms contributing to the pathogenesis of APN include *Citrobacter* spp, *Proteus* spp, *Klebsiella* spp, and enterococci [3, 4].

Uropathogenic *E*. *coli* (UPEC) expresses a variety of virulence factors that contribute to its capacity to colonize the urinary tract and cause disease. The most important include surface-associated factors (flagella, outer-membrane vesicles, pili, curli, non-pilus adhesins, polysaccharide capsules, outer-membrane proteins, and lipopolysaccharide), toxins (α-hemolysin and cytotoxic necrotizing factor type 1), and iron-acquisition systems [5]. Several studies have shown that virulence factors do not act individually but in a coordinated way to guarantee the successful survival and persistence of UPEC in the hostile environment of the urinary tract [6–8].

Since a strain’s propensity to cause disease varies with its phylogenetic origins, one needs rapid and universal tools to identify the clones/clonal complexes/phylogroups of *E*. *coli*. MLST provides the best means of typing *E*. *coli*, although the knowledge of ST does not directly provide any information regarding strain’s phylogroup. Clermont et al. [9] proposed a successful phylogroup assignment based on the presence/absence of *chuA* and *yjaA* genes, and one DNA fragment (TspE4.C2). This method allows to classify *E*. *coli* under four main phylogroups (A, B1, B2 and D) whose assignments are highly congruent with those derived from MLST data [10].

Although different studies analyzing the prevalence of many virulence factors in different UTIs have been performed in the past, pyelonephritis-associated virulence factors have not yet been clearly defined [11–21].

In this frame, the main aim of the present work is to examine the virulence factor repertoire exhibited by *E*. *coli* strains prospectively isolated from women with community-acquired primitive APN. We hypothesized that primitive APN-associated strains would exhibit a distinct virulence repertoire, and we inferred that any virulence factors, or phylogenetic groups, specifically associated with this cohort should reflect high virulence. For this purpose, broad-range virulence genotyping assays, and amplification-based phylotyping methods were used to characterize 53 *E*. *coli* strains isolated in urine samples from patients with primitive APN, as compared to recurrent cystitis (RC)-associated strains and clinically not significant (colonizers) strains. The associations of virulence factor genes with regard to patient status, phylogenetic background, antibiotic resistance, motility, and biofilm formation were also investigated.

## Materials and methods

### Study subjects

A total of 53 patients were enrolled in this prospective study over a two-year period (May 2012 to May 2014). Subjects were aged from 18 to 52 years, with a median age of 30 years.

Eighteen consecutive patients were assigned a diagnosis of primitive (uncomplicated) APN based on the following criteria: i) at least one of the following symptoms was observed: temperature ≥ 38°C, urgency, frequency, dysuria or suprapubic tenderness; and ii) quantitative midstream urine culture yielded bacterial growth ≥ 10^5^ colony-forming units (CFU)/mL (or > 10^4^ CFU/mL when antibiotic therapy was started before collecting urine) [22]. The presence of systemic and/or anatomical predisposing factors, and/or immune-impairment or pregnancy, defined the APN as “complicated” or secondary, excluding such patients from enrollment.

Nineteen patients were diagnosed for RC, defined as three or more uncomplicated UTIs (minimum concentration of 10^5^ CFU/ml in midstream urine, dysuria, pollakiuria, lower abdominal pain, urinary urgency, pyuria) in the last 12 months. Both APN and RC infections were considered community-acquired since patients were diagnosed within the first 48 h of hospitalization.

A total of 16 volunteer subjects having normal urinalysis, a negative urine culture (< 10^3^ CFU/mL), and no history of UTI served as controls. Both RC and control (CO) groups were age-matched with the APN group.

The Ethical Committee of the “G. d’Annunzio” University of Chieti-Pescara confirmed that ethical approval was not needed since the study deals with a non-invasive diagnostic tool used according to standard indications in a specific subset of patients, for which previous indications are available in the literature. As clinical information for patients with UTI was collected anonymously, patient consent was not obtained.

### Clinical and reference strains

Fifty-three *E*. *coli* strains were prospectively collected in the Laboratory of Clinical Microbiology at “G. d’Annunzio” University of Chieti-Pescara, Italy: 18 from APN patients; 19 from patients with RC, and 16 “colonizer” apathogenic strains isolated from healthy subjects, namely those without signs or symptoms of genitourinary tract disease (controls, CO). Each strain was isolated from freshly voided midstream urine cultures, and identified by biochemical profiling using the Vitek-2 system (bioMérieux, Marcy l'Etoile, France). Only patients with *E*. *coli* as the sole positive isolate were included in the study. One arbitrarily chosen *E*. *coli* colony per specimen was studied. One arbitrarily chosen *E*. *coli* colony per specimen was selected from the initial culture plate and stored at −80°C (Microbank; ProLab Diagnostics, Wirral, UK) until used.

Other strains used as controls for specific virulence factor gene detection were kindly provided by James R. Johnson (University of Minnesota, Minneapolis, USA) and included: J96 (*papA*, *papC*, *papEF*, *papG* alleles I and III, *sfa/foc*, *focG*, *kpsMT* III, *hlyA*, *cnf1*, *rfc)*, IA2 *(papG* allele II), LG1315*(iutA)*, P678-54/pHK1 1 *(cvaC)*, DH5a/pCIB10B *(ibeA)*, IH11165 *(bmaE*, *gafD)*, HB101/pSR366 *(kpsMT* II), P678-54/pAH1010 *(nfaE)*, E6468/62 *(cdtB)*, P678-54/pKT107 *(traT)*, 536-21/pANN801 13 *(sfa/foc*, *sfaS)*, NS24 (K1), GR12 (K5), and A30 *(afa/dra*) [19]. Strain P678-54 was used as a negative control [11].

### Clonal relatedness and phylogenetic analysis

Relatedness among isolates was established by Pulsed Field Gel Electrophoresis (PFGE) analysis. Agarose-embedded chromosomal DNA was digested with the restriction enzyme *XbaI*, and the fragments obtained were separated in a CHEF MAPPER XA apparatus (Bio-Rad; Hercules, CA). The DNA banding patterns were normalized with bacteriophage lambda concatemer ladder standard (Bio-Rad) and PFGE patterns were compared using the criteria for strain relatedness established by Tenover et al. [23].

Phylogenetic grouping into groups A, B1, B2, and D was determined by a triplex PCR assay using *chuA*, *TspE4*.*C2* and *yjaA* as DNA markers [9]. PCR conditions were as follows: activation for 10 min at 95°C, 30 cycles of 5 s at 95°C, 10 s at 59°C, and a final extension step of 7 min at 72°C. *E*. *coli* ATCC 25922 was used as a positive control for the B2 phylogenetic group which had all 3 of the DNA markers.

### Detection of virulence factor genes (VFGs)

Each strain was screened by PCR analysis [11, 12, 19, 24] for the presence of the following 28 VFGs, presumptively associated with extraintestinal pathogenic *E*. *coli* (S1 Table): i) adhesins: *papAH* (P fimbriae), *papG* (P fimbria adhesin—alleles I, II and III), *sfa/focDE* (S and F1C fimbriae), *focG* (F1C fimbrial adhesin), *iha* (iron-regulated gene homologue adhesin), *afa/draBC* (Dr family adhesin), *bmaE* (M fimbriae), *gafD* (G fimbriae), *nfaE* (nonfimbrial adhesin), *sfaS* (S fimbrial adhesin), and *ecpA* (*E*. *coli* common pilus); ii) siderophores: *iutA* (aerobactin receptor), *fyuA* (ferric yersiniabactin receptor), and *iroN* (catecholate siderophore receptor); iii) toxins: *hlyA* (hemolysin), *cnf1* (cytotoxic necrotizing factor), and *cdtB* (cytolethal distending toxin); iv) capsule: *kpsMT*-II and *kpsMT*-III (group 2 and 3 capsule synthesis), *kpsMT*-*K1* (K1 capsule synthesis); and v) miscellaneous: *rfc* (O4 lipopolysaccharide synthesis), *cvaC* (colicin V), *traT* (serum resistance), *ibeA* (invasion of brain epithelium), *usp* (uropathogenic-specific protein, bacteriocin), *omptT* (outer membrane protein T, protease), and *malX* (a marker for a pathogenicity-associated island, PAI, from archetypal uropathogenic strain CFT073).

The aggregate VFG score was defined as the total number of VFGs detected for a given isolate [11]. VFG patterns were determined as all unique virulence-associated gene assemblages in the study population. Such molecular characteristics were shown to predict experimental virulence *in vivo* [25].

### Antibiotic susceptibility testing

Antimicrobial susceptibility tests were performed by the disk diffusion agar test, according to EUCAST recommendations and interpretative criteria [26], for ampicillin, amoxicillin/clavulanate, piperacillin/tazobactam, cefotaxime, ceftazidime, cefepime, ertapenem, imipenem, meropenem, amikacin, gentamicin, fosfomycin, nitrofurantoin, and cotrimoxazole. The presence of extended spectrum beta-lactamases (ESBL) was assessed using the Vitek-2 ESBL test [27]. *E*. *coli* ATCC25922 was used as a quality control strain. Strains were defined as multi-drug resistant (MDR) when exhibiting resistance to at least one agent in three or more antimicrobial categories [28].

### Biofilm formation

The ability of 53 *E*. *coli* strains to form biofilm was evaluated in a 96-well flat-bottom polystyrene tissue-culture microtiter plate (BD Italia), as previously described [29]. Briefly, each well was inoculated with 200 μL of a standardized suspension (1–3 × 10^7^ CFU/ml) prepared in Tryptone Soya broth (Oxoid S.p.A, Milan, Italy). Following incubation at 37°C for 24 h non-adherent bacteria were removed by three washes with sterile PBS, then biofilm samples were quantified by staining with crystal violet, and finally destained with 33% glacial acetic acid. Biofilm biomass was evaluated by measuring the optical density at 492 nm (OD_492_).

Considering a low cut-off (ODc) represented by 3×SD above the mean OD of control wells (not seeded with bacteria), strains were classified into the following categories [30]: no biofilm producer (OD ≤ ODc), weak biofilm producer (ODc < OD ≤ 2 × ODc), moderate biofilm producer (2 × ODc < OD ≤ 4 × ODc), and strong biofilm producer (4 × ODc < OD).

### Motility assays

Swimming, swarming, and twitching motilities were evaluated as previously described [31], with some modifications. Briefly, individual colonies from an agar growth were transferred, using a sterile needle, to the surface of swimming agar (10 g/l tryptone, 5 g/l NaCl, 0.3% agar) and swarming agar (8 g/l nutrient broth, 5 g/l glucose, 0.5% agar). Swarm plates were allowed to dry overnight at room temperature before being used. After incubation for 48 h, results were expressed as the diameter (mm) of the halo of growth formed around the point of inoculation. Twitching agar (10 g/l tryptone, 5 g/l yeast extract, 10 g/l NaCl, 1% agar) was inoculated into the bottom of the Petri dish plate using a sterile needle. After incubation for 48 h, the diameter (mm) of migration and growth at the agar/Petri dish interface was measured by staining with crystal violet.

### Statistical analysis

Each assay was performed in triplicate and repeated on at least two different occasions. All statistical tests were performed using GraphPad software, version 6.0 (GraphPad Inc.).

Comparisons of proportions were evaluated using the chi-square test or Fisher’s exact test as appropriate. Comparisons between groups were evaluated by parametric (Analysis-of-Variance followed by Tukey’s multiple comparison post-test) or non-parametric (Kruskal-Wallis + Dunn’s multiple comparison post-test, Mann-Whitney test) tests. The D'Agostino & Pearson omnibus normality test was used to assess if the values came from a Gaussian distribution. Correlation between groups was evaluated by calculating the Spearman’s correlation coefficient. Factor analysis using the principal component method (Principal Component Analysis) was performed on detection frequencies of gene presence to identify VFGs that were independently predictive of specific epidemiologic subgroups (APN, RC, and CO). The Principal Component Analysis scores were calculated using the covariance matrix, and the principal components 1 and 2 (PC1 and PC2) were determined to explain most data variation, using a Scree test. PC1 was plotted against PC2 to identify the genes driving the two components and to explain the percentage variation.

For all tests, the threshold for statistical significance was set at *p* < 0.05.

## Results

### Clonal relatedness and phylogenetic analysis

**Fig 1:**
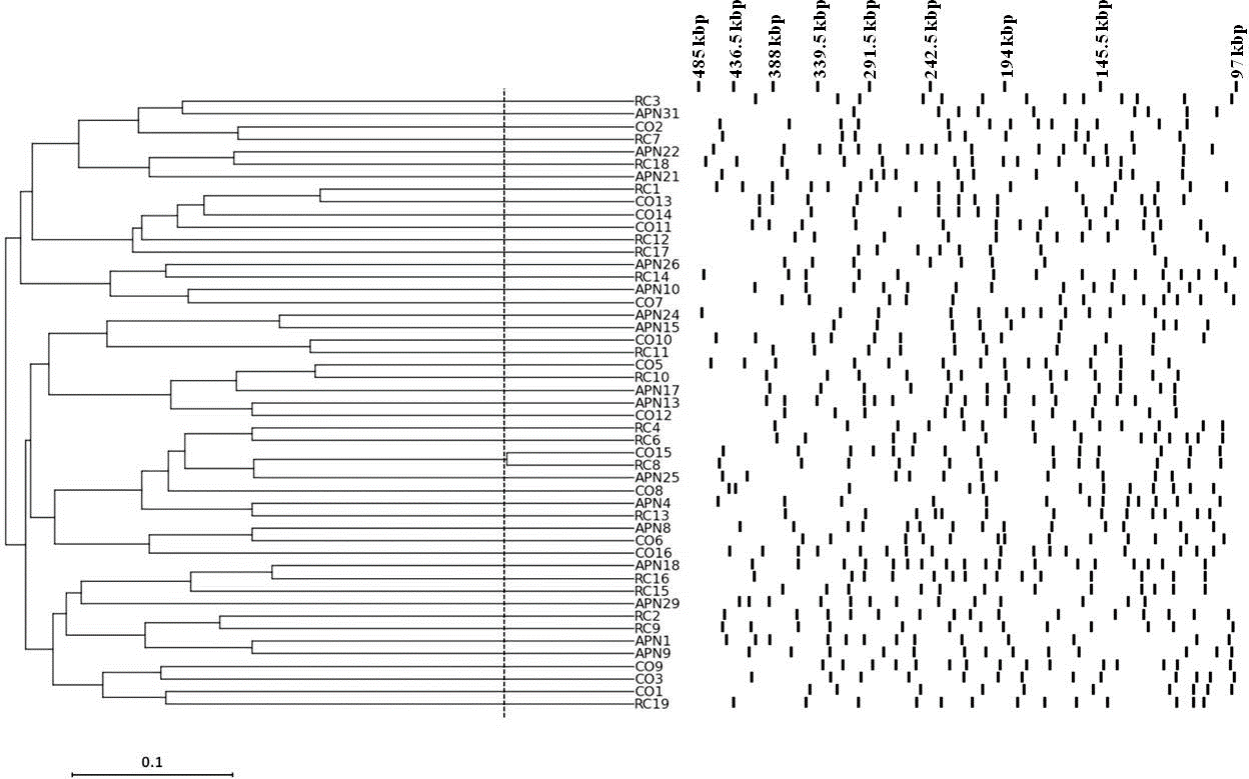
Clonal relatedness. The clonal distribution of 53 *E*. *coli* strains—isolated from women with acute pyelonephritis (APN), recurrent cystitis (RC), or not symptomatic (healthy controls; CO)—was assessed by PFGE analysis. The vertical dotted line shows the three-band-difference breakpoint [23]. Four strains (APN3, APN34, CO4, and RC5) proved not to be typeable by *XbaI* macrorestriction analysis.

The clonal relatedness of *E*. *coli* strain populations was assessed by PFGE analysis, and the results are shown in Fig 1. Tenover et al. [23] defined two strains with identical PFGE profiles as belonging to the same clone, and two strains showing less than or equal to three differences in the PFGE banding patterns as strictly related. If the number of differences was >3, strains might be considered as unrelated. Overall, our findings showed an extremely heterogeneous population with a highly polyclonal distribution. The most genetically related strains, albeit not belonging to the same clone, were CO15 and RC8, showing three differences in the banding pattern.

Phylogenetic group analysis was carried out by triplex PCR assay, and the results are shown in Table 1. The most prevalent phylogenetic group was B1, both considering strains as a whole (37 out of 53, 69.8%; *p*<0.01 vs other groups), and within each study-group (61.1, 78.9, and 68.7% for APN, RC, and CO strains, respectively). APN strains showed unique features, namely the presence of phylogroup A (5 out of 18, 27.8%; *p* = 0.005 vs other groups), and the absence of both phylogroup B2 and non-typeable strains. A total of 3 (5.6%) *E*. *coli* strains were classified as non-typeable.

**Table 1:** Distribution of phylogenetic groups, according to clinical syndrome.

	No. (%) of strains				
Phylogenetic group	APN	RC	CO	*p*-value	Total n (%)
A	5 (27.8)	-	-	**0.005**	5 (9.4)
B1	11 (61.1)^a^	15 (78.9)^a^	11 (68.7)^a^	0.495	37 (69.8)^a^
B2	-	2 (10.5)	1 (6.3)	0.380	3 (5.7)
D	2 (11.1)	1 (5.3)	2 (12.5)	0.733	5 (9.4)
NT	-	1 (5.3)	2 (12.5)	0.288	3 (5.7)

*E*. *coli* strains were isolated from women with acute pyelonephritis (APN; n = 18), recurrent cystitis (RC; n = 19), or not symptomatic (healthy controls; CO; n = 16). Phylogenetic groups were evaluated using the PCR triplex assay, according to Clermont et al. [9]: A (n = 5), B1 (n = 37), B2 (n = 3), D (n = 5). NT (n = 3), non-typeable. Bold values are statistically significant (*p*<0.05) as assessed by the chi-square test.

^a^ *p*<0.01 vs other phylogenetic groups, chi-square test.

### VFGs distribution by source and phylogenetic group

The presence of 28 VFGs in each strain was evaluated by PCR, the results being summarized in Table 2. Considering the strains as a whole, the most dominant VFGs were *ecpA* and *fyuA*, found in a significantly higher percentage of strains (92.4 and 86.7%, respectively) than other VFGs screened (*fyuA* vs *papG II/III*, *p*<0.05; *fyuA* vs other VFGs, *p*<0.01). Particularly, these genes along with *PapG II/III*, and *hlyA* were found in at least 50% of strains, whereas *bmaE*, *gafD*, *nfaE*, *cdtB*, *kpsMT-III*, and *ompT* were always lacking.

**Table 2:** Distribution of virulence factor genes, according to clinical syndrome.

	No. (%) of strains				*p*-value		
Virulence genes	Total (n = 53)	APN (n = 18)	RC (n = 19)	CO (n = 16)	APN vs RC	APN vs CO	RC vs CO
**Adhesins**							
*ecpA*	49 (92.4)	17 (94.4)	18 (94.7)	14 (87.5)	1.000	0.591	0.446
*papG II/III*	35 (66.0)	15 (83.3)	11 (57.9)	9 (56.3)	0.151	0.134	0.922
*iha*	20 (37.7)	12 (66.7)	6 (31.6)	2 (12.5)	**0.045**	**0.002**	0.243
*sfa/focDE*	19 (35.8)	5 (27.8)	10 (52.6)	4 (25.0)	0.184	1.000	0.096
*sfaS*	16 (30.1)	5 (16.7)	6 (31.6)	5 (31.3)	0.447	0.429	0.983
*focG*	13 (24.5)	3 (16.7)	5 (26.3)	5 (31.3)	0.693	0.429	0.748
*afa/draBC*	5 (9.4)	4 (16.7)	-	1 (6.3)	0.105	0.604	0.269
*papAH*	5 (9.4)	3 (16.7)	2 (10.5)	-	0.660	0.230	0.489
*papG I*	2 (3.7)	2 (11.1)	-	-	0.230	0.487	
*bmaE*	-	-	-	-			
*gafD*	-	-	-	-			
*nfaE*	-	-	-	-			
**Siderophores**							
*fyuA*	46 (86.7)	17 (94.4)	19 (100.0)	10 (62.5)	0.486	**0.035**	**0.003**
*iroN*	30 (56.6)	8 (44.4)	16 (84.2)	6 (37.5)	**0.016**	0.738	**0.004**
*iutA*	25 (47.1)	12 (66.7)	7 (36.8)	6 (37.5)	0.103	0.168	0.968
**Toxins**							
*cnf1*	33 (62.2)	8 (44.4)	15 (78.9)	10 (62.5)	**0.045**	0.327	0.283
*hlyA*	32 (60.3)	9 (50.0)	15 (78.9)	8 (50.0)	0.091	1.000	0.072
*cdtB*	-	-	-	-			
**Capsule**							
*kpsMT-II*	31 (58.4)	9 (50.0)	16 (84.2)	6 (37.5)	**0.038**	0.510	**0.004**
*kpsMT-K1*	7 (13.2)	4 (22.2)	1 (10.5)	2 (12.5)	0.405	0.660	0.855
*kpsMT-III*	-	-	-	-			
**Miscellaneous**							
*traT*	26 (49.0)	8 (44.4)	12 (63.2)	6 (37.5)	0.330	0.738	0.130
*malX*	21 (39.6)	9 (44.4)	12 (57.9)	-	0.517	**0.003**	**0.001**
*usp*	21 (39.6)	8 (44.4)	11 (57.9)	2 (12.5)	0.517	**0.043**	**0.006**
*ibeA*	5 (9.4)	1 (5.6)	1 (5.3)	3 (18.8)	1.000	0.323	0.212
*rfc*	2 (3.7)	-	2 (10.5)	-	0.486		0.156
*cvaC*	1 (1.8)	-	1 (5.3)	-	1.000		0.402
*omptT*	-	-	-	-			

Twenty-Eight *E*. *coli* virulence factor genes tested were screened by PCR. See Materials and Methods for gene description. Bold values are statistically significant (*p*<0.05) as assessed by the chi-square test or Fisher’s exact test, when appropriate.

With regard to the three source groups, APN and RC strains showed a significantly higher prevalence of *fyuA*, *usp*, and *malX* than did control strains. Compared to RC strains, APN showed a lower prevalence of *iroN*, *cnf1*, and *kpsMT-II*, but a higher prevalence of *iha*. *PapG I* was found in two APN strains alone, while *rfc* and *cvaC* were respectively observed only in two and one RC strains. *papAH* and *malX* were exclusive for uropathogenic APN and RC strains, having been found in at least 10.5% and 44.4% of strains, respectively. The only strain that proved negative for all VFGs screened was CO4.

**Fig 2:**
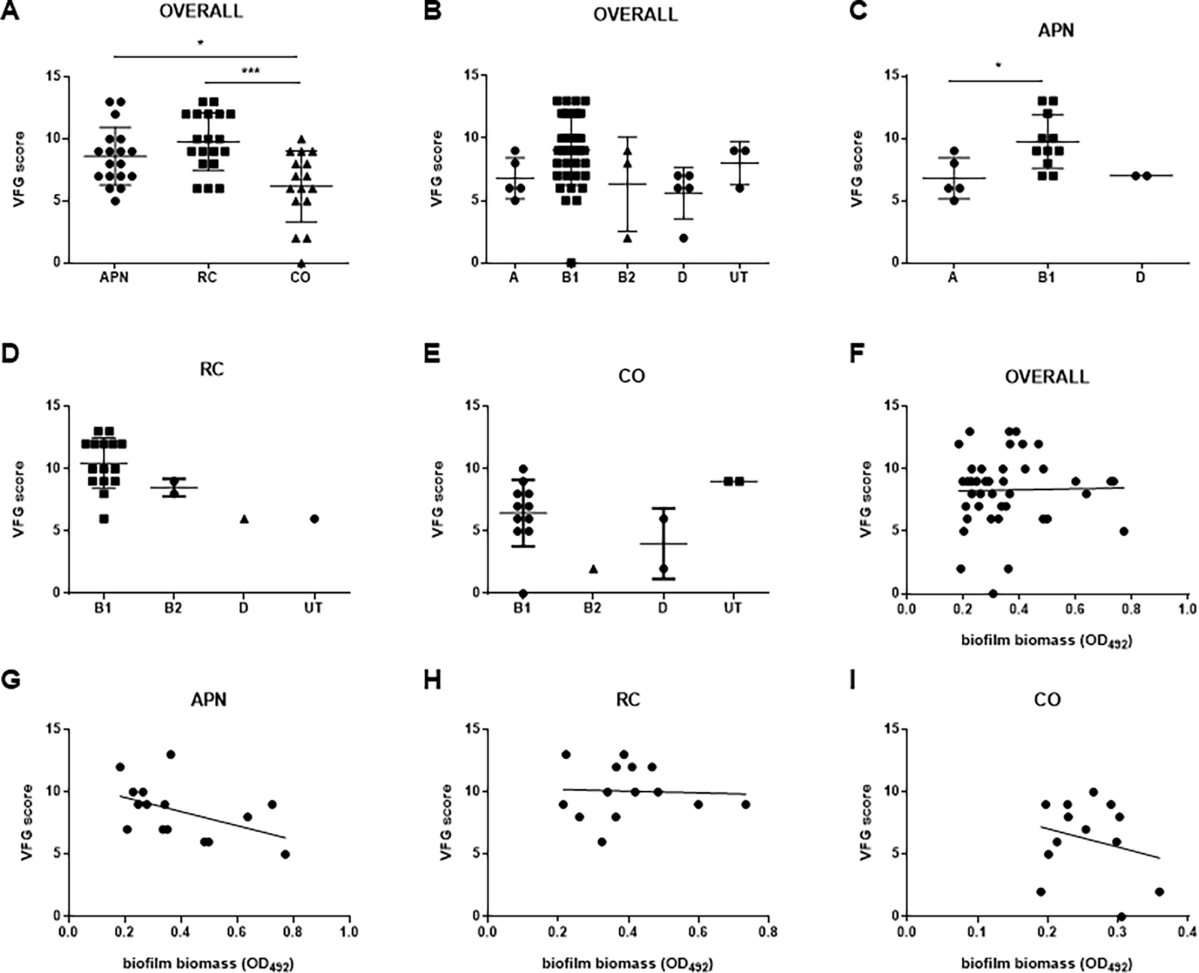
Virulence factor gene (VFG) score. **A**) Distribution of VFG scores among *E*. *coli* strains isolated from women with acute pyelonephritis (APN, n = 18), recurrent cystitis (RC, n = 19), or not symptomatic (healthy controls; CO, n = 16), and **B-E**) according to phylogenetic group, considering strains as a whole or according to clinical syndrome. Results are expressed as a scatter plot, showing mean ± SD. *p<0.05, *** *p*<0.001, ANOVA + Tukey’s multiple comparison post-test. Each dot is the average from three independent experiments with three replicates of each strain per experiment. **F-I**) Correlation between biofilm biomass formed and VFG score, considering all strains as a whole or according to clinical syndrome. Spearman r coefficient calculation showed no significant trends, although a clear trend towards a negative relationship was observed for APN isolates (Spearman r: -0.501; *p* = 0.0508). Each dot is the average from three independent experiments with three replicates of each strain per experiment (n = 9).

For each isolate, the aggregate VFG score—defined as the total number of VFGs detected–was calculated since it predicts experimental virulence *in vivo* [25] (Fig 2). Overall, the aggregate VFG scores extensively varied among the strains tested, ranging from 0 to 13. The mean VFG score exhibited by APN and RC strains was significantly higher than CO ones (mean ± SD: 8.6 ± 2.3 and 9.7 ± 2.3 vs 6.1 ± 2.8, respectively; *p*<0.05 and *p*<0.001, APN vs CO, and RC vs CO, respectively) (Fig 2A). This is because, apart from *ecpA* (87.5%), only four genes (*papG II/III*, *fyuA*, *hlyA*, and *cnf1*) occurred in at least 50% of CO isolates. No significant differences among phylogroups were observed in the VFG score (Fig 2B).

To investigate the relationship between phylogenetic groups and VFGs, the VFG score was calculated according to the phylogroups, the results being summarized in Fig 2B–2E. Considering APN strains, the VFG score shown by strains belonging to phylogroup B1 was significantly higher than phylogroup A (VFG score, mean ± SD: 9.7 ± 2.1 vs 6.8 ± 1.6, respectively; *p*<0.05) (Fig 2C).

With regard to each VFG tested, significant differences in prevalence between study-groups were found for B1 strains alone (Table 3): *iha* was significantly more prevalent in APN strains than in CO ones (63.6 vs 0%, respectively; *p*<0.05), *fyuA* and *malX* were significantly more prevalent in APN and RC strains (*fyuA*: 100 vs 100 and 63.6%; *malX*: 73.3 and 63.6 vs 0%; respectively for RC, APN, and CO; *p*<0.01), whereas *kpsMT-II* was significantly more dominant in RC strains (*kpsMT-II*: 93.3 vs 54.5 and 36.3%, respectively for RC, APN, and CO; *p*<0.01). Within each study-group, no significant differences were found among phylogroups. On the other hand, the prevalence of some VFGs observed in strains belonging to phylogroup B1 significantly differed between study groups (Table 3): *iha* (APN > others; *p* = 0.006), *fyuA* (APN and RC > CO; *p* = 0.005), *kpsMT*-*II* (RC > others; *p* = 0.008), and *malX* (RC > others; *p*<0.001). It is worth noting that *focG* and *sfaS* genes were confined to phylogroup B1.

**Table 3:** Virulence factor gene distribution, according to phylogenetic group.

	No. (%) of strains								
	APN			RC			CO		
Virulence genes	A (n = 5)	B1 (n = 11)	D (n = 2)	B1 (n = 15)	B2 (n = 2)	D (n = 1)	B1 (n = 11)	B2 (n = 1)	D (n = 2)
**Adhesins**									
*papAH*	-	2 (18.1)	1 (50.0)	2 (13.3)	-	-	-	-	-
*papG* II/III	5 (100)	8 (72.7)	2 (100)	8 (53.3)	2 (100)	1 (100)	7 (63.6)	-	-
*papG* I	-	1 (9)	-	-	-	-	-	-	-
*sfa/focDE*	1 (20.0)	4 (36.3)	-	8 (53.3)	2 (100)	-	3 (27.2)	-	-
*focG*	-	3 (27.2)	-	5 (33.3)	-	-	5 (45.4)	-	-
*iha*	3 (30.0)	**7 (63.6)**^a^	2 (100)	5 (33.3)	-	1 (100)	-	1 (100)	-
*afa*/*draBC*	-	3 (27.2)	-	-	-	-	-	-	1 (50.0)
*bmaE*	-	-	-	-	-	-	-	-	-
*gafD*	-	-	-	-	-	-	-	-	-
*nfaE*	-	-	-	-	-	-	-	-	-
*sfaS*	-	3 (27.2)	-	6 (40.0)	-	-	4 (36.3)	-	-
*ecpA*	4 (80.0)	11 (100)	2 (100)	15 (100)	2 (100)	1 (100)	10 (90.9)	-	2 (100)
**Siderophores**									
*iutA*	4 (80.0)	6 (54.5)	2 (100)	5 (33.3)	-	1 (100)	3 (27.2)	1 (100)	1 (50.0)
*fyuA*	4 (80.0)	11 (100)	2 (100)	15 (100)	2 (100)	1 (100)	**7 (63.6)**^b^	-	2 (100)
*iroN*	1 (20.0)	7 (63.6)	-	13 (86.6)	2 (100)	-	5 (45.4)	-	-
**Toxins**									
*hlyA*	2 (40.0)	7 (63.6)	-	13 (86.6)	2 (100)	-	7 (63.6)	-	-
*cnf1*	2 (40.0)	5 (45.4)	-	13 (86.6)	2 (100)	-	8 (72.7)	-	-
*cdtB*	-	-	-	-	-	-	-	-	-
**Capsule**									
*kpsMT-II*	1 (20.0)	6 (54.5)	2 (100)	**14 (93.3)**^c^	1 (50.0)	-	4 (36.3)	-	1 (50.0)
*kpsMT-III*	-	-	-	-	-	-	-	-	-
*kpsMT-K1*	1 (20.0)	3 (27.2)	-	2 (13.3)	-	-	1 (9.0)	-	-
**Miscellaneous**									
*rfc*	-	-	-	-	2 (100)	-	-	-	-
*cvaC*	-	-	-	-	-	-	-	-	-
*traT*	3 (60.0)	4 (36.3)	1 (50.0)	10 (66.6)	-	1 (100)	4 (36.3)	-	1 (50.0)
*ibeA*	-	1 (9.0)	-	1 (6.6)	-	-	3 (27.2)	-	-
*usp*	1 (20.0)	7 (63.6)	-	11 (73.3)	-	-	-	-	-
*omptT*	-	-	-	-	-	-	-	-	-
*malX*	1 (20.0)	**7 (63.6)**^d^	-	**11 (73.3)**^d^	-	-	-	-	-

*E*. *coli* strains were isolated from women with: primitive acute pyelonephritis (APN, n = 18); recurrent cystitis (RC, n = 19); no symptomatology (controls, CO, n = 16). Phylogenetic groups were evaluated using PCR triplex assay, according to Clermont et al. [9]: A (n = 5), B1 (n = 37), B2 (n = 3), D (n = 5). No significant differences were found between phylogroups, within each study-group. Bold values are significant (*p*<0.05) differences found between study-groups as assessed by the chi-square test or Fisher’s exact test, when appropriate

^a^ APN vs CO (*p* = 0.006)

^b^ CO vs APN and RC (*p* = 0.005)

^c^ RC vs APN and CO (*p* = 0.008)

^d^ APN and RC vs CO (*p*<0.01).

Forty-nine unique VFG assemblages were observed, with only four shared by two strains each: two assemblages shared by strains belonging to RC groups (RC2 and RC9; RC4 and RC5), whereas the remaining two were shared by strains belonging to different groups (APN21 and RC16; APN29 and RC17) (data not shown).

**Fig 3:**
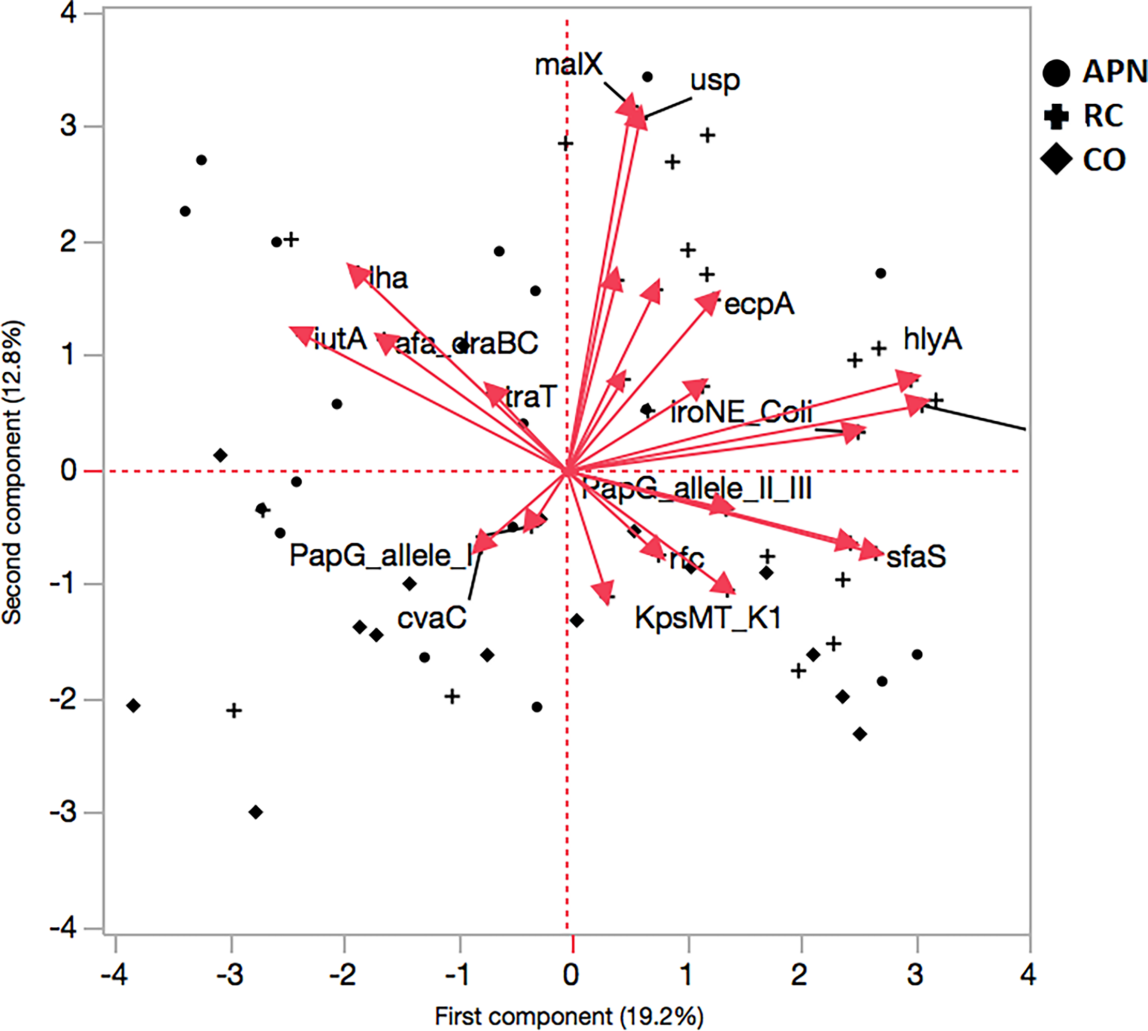
Principal component analysis based on VFG detection. Three groups of subjects were enrolled: women with acute pyelonephritis (APN, n = 18), recurrent cystitis (RC, n = 19), or not symptomatic healthy controls (CO, n = 16). PC1 represent 19.2% of the data variability, whereas PC2 represents 12.8% of such variability.

Principal component (PC) analysis, based on the presence of VFGs, clustered *E*. *coli* strains into two groups that incorporate 32% cumulative variation: PC1 explained 12.8% of the variation, whereas PC2 explained 12.8% of the variation (Fig 3). Plotting PC1 against PC2 for visual evaluation revealed trends in differentiation and clustering of groups with a similar gene pattern.

Several different genes, such as *hlyA* and *iutA* loaded heavily on PC2, whereas *malX*, *usp* and *fyuA* determined the loading for PC1 (Fig 3).

The gene clusters derived from hierarchical cluster analysis were based on their contribution to cluster formation (Table 4). In cluster 1, the most representative genes *hlyA* and *cnf1* were detected in each group with comparable frequencies. In cluster 2, the most representative genes *malX* and *usp* were detected in a significantly higher percentage in the APN and RC groups than in CO (44.4 and 57.9% vs 12.5%, respectively; *p* = 0.025). Furthermore, no CO strains simultaneously showed both genes. In cluster 3, *iutA* and *iha* were detected more frequently in APN strains (78.0%; *p* = 0.003). In cluster 4, the most representative genes *fyuA* and *ecpA* were found more frequently in RC and APN than in CO strains (100 and 94.4% vs 87.5%, respectively; *p* = 0.042).

**Table 4:** Virulence factor gene cluster distribution.

Cluster	No. of genes	Most representative genes	Cluster proportion of variance explained	Total proportion of variation explained	Percentage of strains belonging to:			*p*-value
1	5	*hlyA*, *cnf1*	0.569	0.129	44.4	78.9	50.0	0.150
2	4	*malX*, *usp*	0.529	0.096	44.4	57.9	12.5	**0.025**^a^
3	6	*iutA*, *iha*	0.438	0.120	55.5	31.6	12.5	**0.003**^b^
4	2	*fyuA*, *ecpA*	0.761	0.069	94.4	94.7	62.5	**0.042**^c^

VFGs clusters were determined by Principal Component Analysis based on the detection frequency of virulence genes in women with acute pyelonephritis (APN, n = 18), recurrent cystitis (RC, n = 16), or not symptomatic (healthy controls; CO, n = 19). The first principal component (PC1) of genes in each cluster was considered. Most representative clusters were shown. Bold values are statistically significant (*p*<0.05) as assessed by the chi-square test

^a^ APN and RC vs CO

^b^ APN vs RC and CO

^c^ APN and RC vs CO.

### Motility assays

Swimming, swarming, and twitching motilities were assayed, and the results are shown in S1 Fig and Table 5. No significant differences were observed in motility efficiency, as shown by the median motility level, regardless of the study-groups and phylogroups considered (S1 Fig). However, striking differences were observed in the ability for movement among the study-groups considered (Table 5). A significantly higher proportion of swarming-positive strains was observed in APN than in CO strains (83.3 vs 63.1 and 37.5%, respectively; *p*<0.05). All *E*. *coli* strains showed twitching motility, whereas swimming motility was more frequent among CO strains than RC strains (87.5 vs 42.1%, respectively; *p*<0.05). By contrast, no differences were found in the prevalence of motility types among phylogroups (Table 5).

**Table 5:** Distribution of motility, according to clinical syndrome and phylogroup.

	No. (%) of strains positive for:				No. (%) of strains positive for:		
group (n)	swarming	twitching	swimming	phylogroup (n)	swarming	twitching	swimming
APN (18)	15 (83.3)	18 (100)	12 (66.6)	A (5)	4 (80.0)	5 (100)	3 (60.0)
RC (19)	12 (63.1)	19 (100)	8 (42.1)	B1 (37)	24 (64.8)	37 (100)	23 (62.1)
CO (16)	6 (37.5)	16 (100)	14 (87.5)	B2 (3)	1 (33.3)	3 (100)	2 (66.6)
				D (5)	2 (40.0)	5 (100)	4 (80.0)
				NT (3)	3 (100)	3 (100)	2 (66.6)
*p*-value	**0.018**^a^	1.000	**0.019**^b^		0.315	1.000	0.955

Swimming, swarming, and twitching motilities were assessed for 53 *E*. *coli* strains isolated from women with acute pyelonephritis (APN), recurrent cystitis (RC), or not symptomatic (healthy controls; CO). Phylogenetic groups were evaluated using PCR triplex assay according to Clermont et al. [9]: A, B1, B2, D, NT (non-typeable). Bold values are statistically significant (*p*<0.05) as assessed by the chi-square test

^a^ APN vs CO

^b^ RC vs CO.

### Antibiotic resistance

*In vitro* antibiotic susceptibility test results are summarized in Table 6. No statistically significant differences in antibiotic-resistance rates were found. The prevalence of resistance to the tested antibiotics was generally low, ranging from 0% (ertapenem, imipenem, meropenem, and nitrofurantoin) to 18.8% (amoxicillin/clavulanic acid, and cotrimoxazole), except for ampicillin whose resistance rate was 37.7%. Antibiotic resistance profiles were not associated with a specific study- or phylogenetic group. However, a trend suggestive of higher resistance to ampicillin and cotrimoxazole was found in APN strains, although not statistically significant (ampicillin: 44.4 vs 31.6 and 37.5%; cotrimoxazole: 27.8 vs 15.8 and 12.5%; respectively for APN, RC, and CO strains). Resistance to amikacin was observed in strain APN1 alone (5.6%), whereas resistance to piperacillin/tazobactam was found in two RC strains only (RC2 and RC8; 10.5%). Resistance to cephalosporins (cefotaxime, ceftazidime, and cefepime) was observed only in ESBL-positive strain CO7 (6.3%). No other ESBL-positive strains were found.

**Table 6:** *In vitro* susceptibility to antibiotics.

	No. (%) of resistant strains:	No. (%) of resistant strains:			*p*-value	No. (%) of resistant strains:				*p*-value
Antibiotics		APN	RC	CO		A	B1	B2	D	
Ampicillin	20 (37.7)	8 (44.4)	6 (31.6)	6 (37.5)	0.722	3 (60.0)	13 (35.1)	-	3 (60.0)	0.348
Amoxicillin/clavulanate	10 (18.8)	3 (16.7)	3 (15.8)	4 (25.0)	0.753	1 (20.0)	8 (21.6)	-	-	0.755
Piperacillin/tazobactam	2 (3.7)	-	2 (10.5)	-	0.156	-	2 (5.4)	-	-	
Cefotaxime	1 (1.8)	-	-	1 (6.3)	0.308	-	-	-	1 (20.0)	
Ceftazidime	1 (1.8)	-	-	1 (6.3)	0.308	-	-	-	1 (20.0)	
Cefepime	1 (1.8)	-	-	1 (6.3)	0.308	-	-	-	1 (20.0)	
Ertapenem	-	-	-	-		-	-	-		
Imipenem	-	-	-	-		-	-	-		
Meropenem	-	-	-	-		-	-	-		
Amikacin	1 (1.8)	1 (5.6)	-	-	0.371	-	1 (2.7)	-	-	
Gentamicin	2 (3.7)	1 (5.6)	-	1 (6.3)	0.556	-	1 (2.7)	-	-	
Ciprofloxacin	5 (9.4)	2 (11.2)	1 (5.3)	2 (12.5)	0.622	2 (5.4)	-	2 (40.0)		0.141
Fosfomycin	1 (1.8)	-	1 (5.3)	-	0.402	-	1 (2.7)	-	-	
Nitrofurantoin	-	-	-	-		-	-	-	-	
Cotrimoxazole	10 (18.8)	5 (27.8)	3 (15.8)	2 (12.5)	0.478	1 (20.0)	6 (16.2)	-	3 (60.0)	0.142
MDR^a^	8 (15.0)	3 (16.6)	2 (10.5)	3 (18.7)	0.755	-	6 (16.2)	-	1 (20.0)	0.902

Susceptibility testing was performed, using the disk diffusion agar method, and interpreted according to EUCAST guidelines [26]. Strains were isolated from women with: primitive acute pyelonephritis (APN, n = 18); recurrent cystitis (RC, n = 19); no symptomatology (controls, CO, n = 16). Phylogenetic groups were evaluated using PCR triplex assay, according to Clermont et al. [9]: A (n = 5), B1 (n = 37), B2 (n = 3), D (n = 5). No significant differences were found by the chi-square test.

^a^ MDR, multi-drug resistant strain.

Regarding phylogroups, higher ampicillin-resistance were observed in phylogroups A and D, and higher resistance to ciprofloxacin and cotrimoxazole in phylogroup D; however, both trends were statistically not significant.

The overall resistance rate, calculated as the ratio between the total number of resistances and the total antimicrobial susceptibility tests performed, was comparable among the groups tested (APN: 18 out of 270, 6.6%; RC: 15 out of 342, 4.3%; CO: 16 out of 288, 5.5%). Similarly, MDR strains–those that are resistant to at least one agent in three or more antimicrobial categories [28]—were observed as having comparable prevalence in each group (APN: 3 out of 18, 16.6%; RC: 2 out of 19, 10.5%; CO: 3 out of 16, 18.7%). Almost all MDR strains belonged to phylogroup B1 (6 out of 7, 85.7%).

No correlation was observed between the number of antibiotic resistances and the VFG score, whether considering strains as a whole, or according to clinical syndrome (S2 Fig).

### Biofilm formation

**Fig 4:**
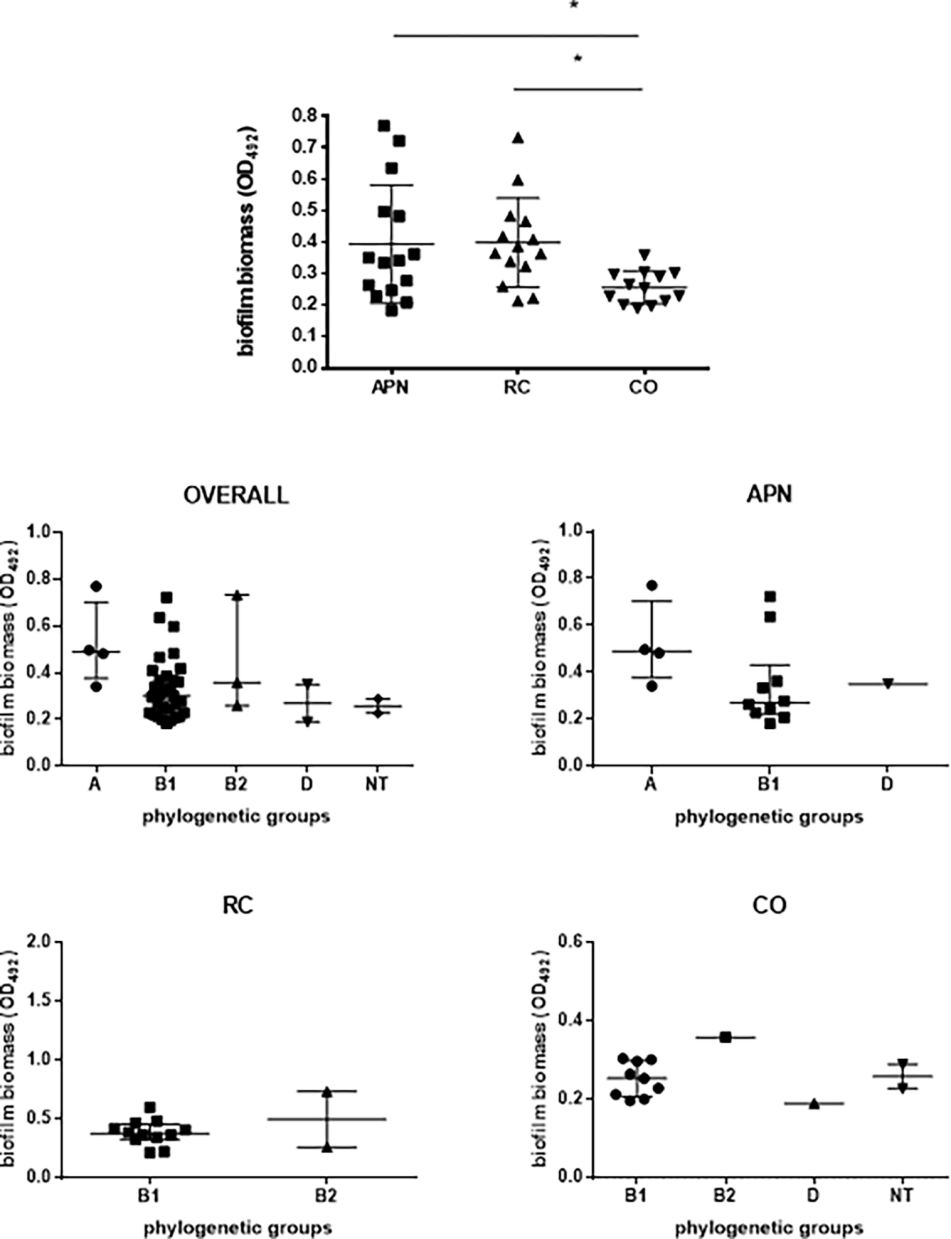
Biofilm formation, according to clinical syndrome and phylogenetic group. Strains were isolated from women with acute primitive pyelonephritis (APN), recurrent cystitis (RC), or healthy subjects (controls, CO). Biofilm forming ability onto polystyrene was spectrophotometrically assessed by crystal violet stain [29]. Phylogenetic groups were evaluated using PCR triplex assay, according to Clermont et al. [9]: A, B1, B2, D, NT (non-typeable). Results are expressed as scatter plots, where bars indicate median values with interquartile range. Each dot is the average from two independent experiments with eight replicates of each strain per experiment (n = 16). Only biofilm-positive strains were considered since “not producer” strains had comparable prevalence both in groups and phylogroups (see Table 7). * *p*<0.05, ANOVA + Tukey’s multiple comparison post-test.

Biofilm forming ability onto polystyrene was spectrophotometrically assessed by crystal violet stain, the results being summarized in Fig 4. Most of the strains tested were able to form biofilm, and no significant differences were found among groups in the proportion of biofilm-positive strains (83.3, 73.6, and 81.2% for APN, RC, and CO, respectively). However, biofilm forming ability varied greatly (OD_492_ range: 0.183–0.771), with value distribution proving more skewed among APN strains, as suggested by the coefficient of variation values (47.8, 35.5, and 20.2%; respectively for APN, RC, and CO strains). The mean biofilm biomass formed by APN and RC strains was comparable (OD_492_, mean ± SD: 0.394 ± 0.188 and 0.399 ± 0.141, respectively), but significantly higher than CO strains (OD_492_: 0.256 ± 0.05; *p*<0.05).

According to the biofilm amount formed, strains were classified into several categories, according to Stepanovic et al. [30], and the results are summarized in Table 7. Most strains were classified as weak-producers, followed by moderate-producers (56.6% vs 20.7%; *p*<0.001). Eleven strains, equally distributed among the study-groups considered, were not able to form biofilm.

**Table 7:** Biofilm formation.

	No (%) of strains:			*p*-value	No (%) of strains:				*p*-value
Biofilm categories	APN	RC	CO		A	B1	B2	D	
Biofilm producer	15 (83.3)	14 (73.6)	13 (81.2)	0.579	4 (80.0)	31 (83.7)	3 (100)	2 (40.0)	0.107
Weak producer	10 (55.6)	8 (42.1)	12 (75.0)	0.148	1 (20.0)	24 (64.9)	1 (33.3)	2 (40.0)	0.233
Moderate producer	4 (22.2)	6 (31.6)	1 (6.3)	0.181	2 (40.0)	7 (18.9)	2 (66.7)	-	0.144
Strong producer	1 (5.5)	-	-	0.371	1 (20.0)	-	-	-	**0.026**
Not producer	3 (16.7)	5 (26.3)	3 (18.8)	0.749	1 (20.0)	6 (16.2)	-	3 (60.0)	0.142

Results from crystal violet stain assay were stratified into four biofilm-producer categories, according to criteria proposed by Stepanovic *et al*. [30]. Strains were isolated from women with: primitive acute pyelonephritis (APN, n = 18); recurrent cystitis (RC, n = 19); no symptomatology (controls, CO, n = 16). Phylogenetic groups were evaluated using PCR triplex assay, according to Clermont et al. [9]: A (n = 5), B1 (n = 37), B2 (n = 3), D (n = 5). Bold values are statistically significant (*p*<0.05) as assessed by the chi-square test.

No significant differences in biofilm category prevalence were found among the study-groups considered. The only strain classified as a strong-producer was APN26, belonging to phylogroup A (mean OD_492_: 0.771) (20 vs 0%, p = 0.026; phylogroup A vs others, respectively).

No significant differences in the aggregate VFG score were found among biofilm categories (data not shown).

### Association between biofilm formation and other phenotypic or genotypic traits

#### Biofilm formation and phylogenetic groups

No statistically significant differences among phylogenetic groups were found in biofilm formation capacity considering the *E*. *coli* groups (AP, RC, CO) (Fig 4).

#### Biofilm formation and VFGs

The mean VFG aggregate score was not associated with the biofilm biomass formed, regardless whether strains were considered as a whole (Fig 2F) or according to clinical syndrome (Fig 2G–2I). However, a clear though not significant trend towards a negative relationship was observed for APN strains (Spearman r: -0.501; *p* = 0.0508) (Fig 2G).

**Fig 5:**
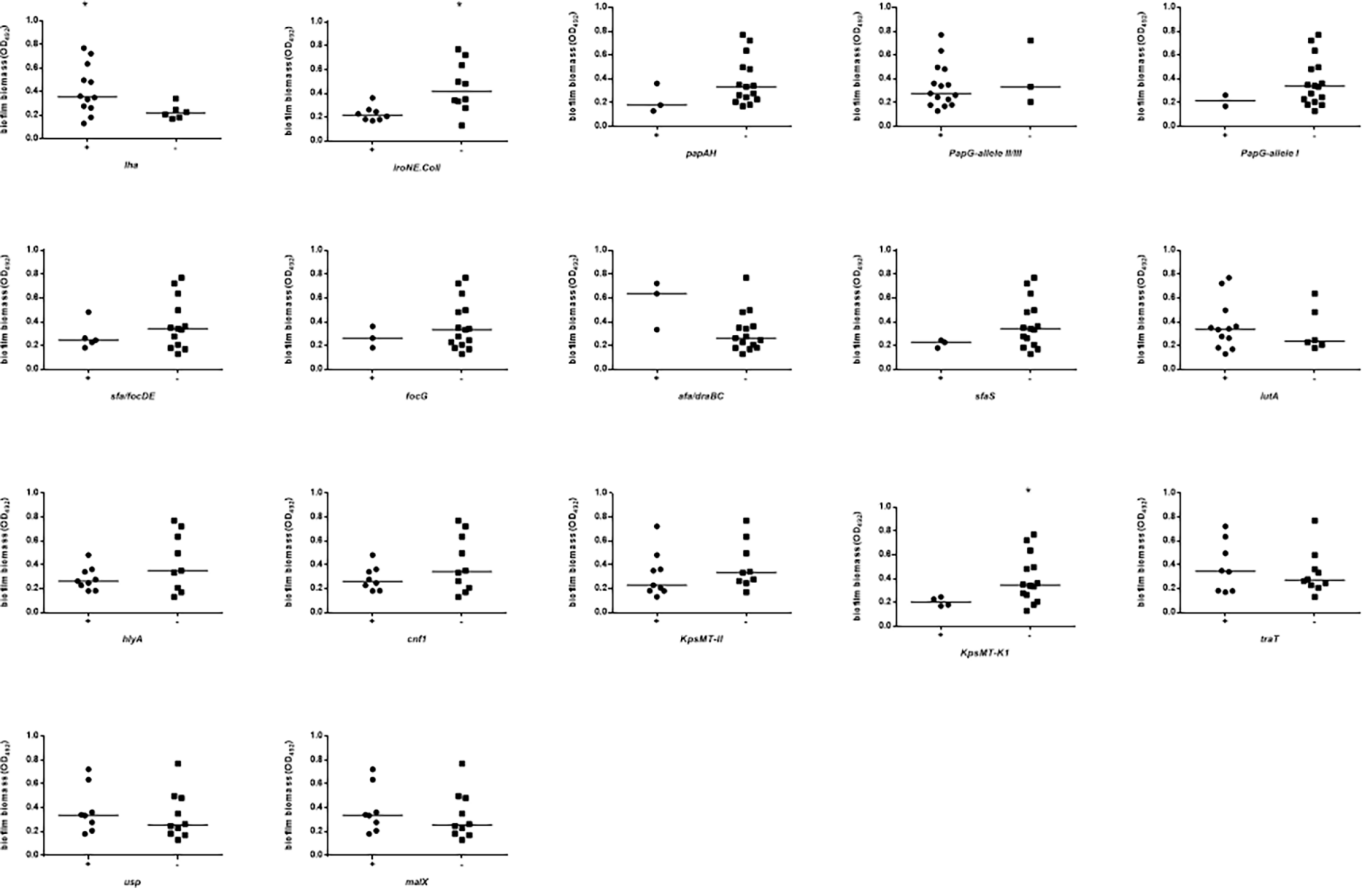
Biofilm formation by *E*. *coli* strains from women with acute pyelonephritis (APN), according to each virulence factor gene (VFG). The presence of VFGs was assessed by PCR. Biofilm forming ability onto polystyrene was spectrophotometrically assessed by crystal violet stain [29]. Each dot is the average from two independent experiments with eight replicates of each strain per experiment (n = 16). Horizontal bars are medians. * *p*<0.05, Mann-Whitney test.

**Fig 6:**
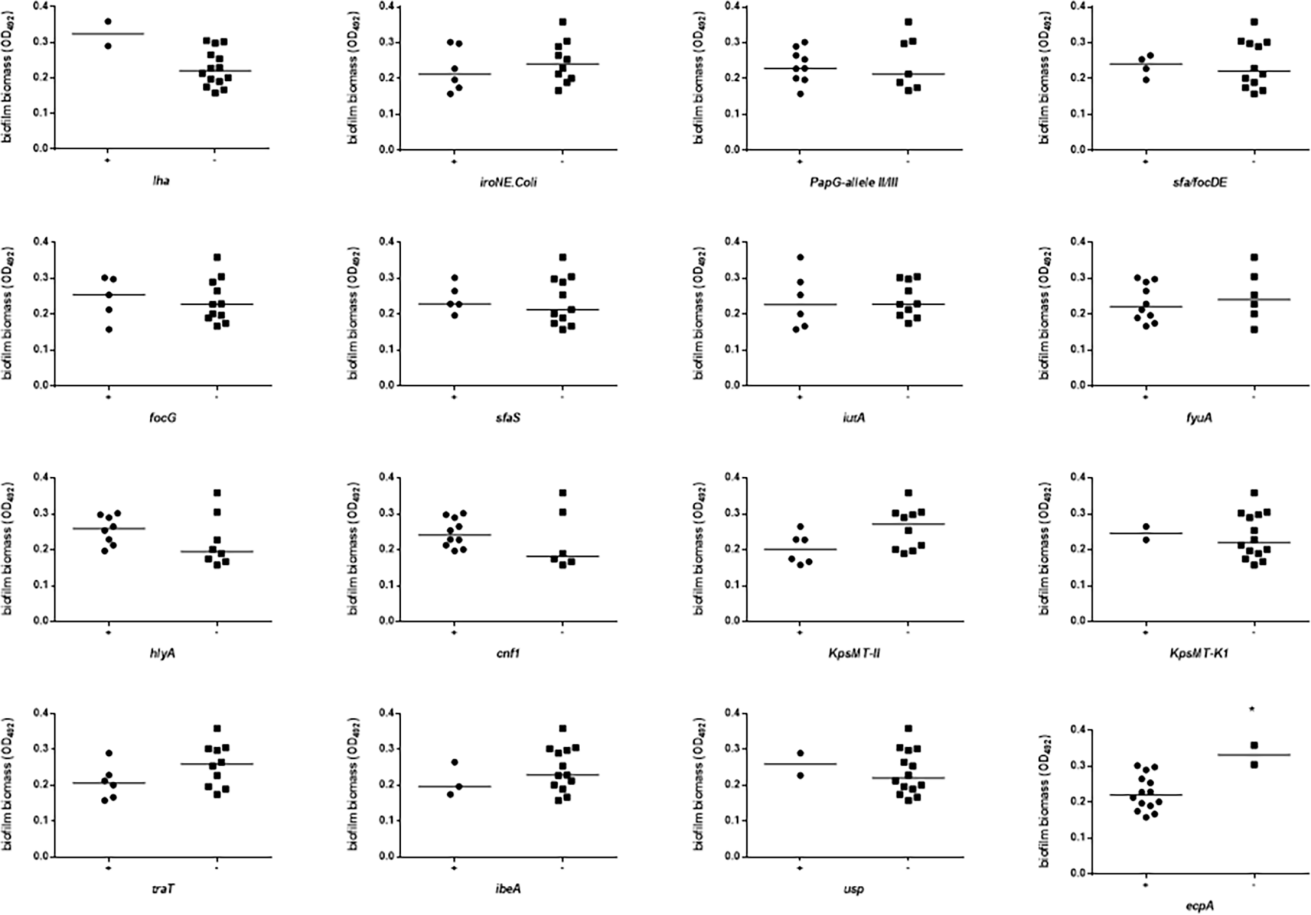
Biofilm formation by *E*. *coli* strains from healthy control women (CO), according to each virulence factor gene (VFG). The presence of VFGs was assessed by PCR. Biofilm forming ability onto polystyrene was spectrophotometrically assessed by crystal violet stain [29]. Each dot is the average from two independent experiments with eight replicates of each strain per experiment (n = 16). Horizontal bars are medians. * *p*<0.05, Mann-Whitney test.

Biofilm biomass was stratified on the presence or absence of each VFG considered, and the results are shown in Figs 5 and 6, and S3 Fig. In APN strains the presence of *iha* was associated with higher biofilm biomass formation (median OD_492_: 0.357 vs 0.218, respectively for *iha*^+^ and *iha*^-^ strains; *p*<0.05), whereas the presence of *iroN* and *KpSMT-K1* was associated with a lower amount of biofilm biomass (median OD_492_: 0.218 vs 0.417, respectively for *iroN*^+^ and *iroN*^-^ strains; *p*<0.05) (median OD_492_: 0.205 vs 0.346, respectively for *KpSMT-K1*^+^ and *KpSMT-K1*^-^ strains; *p*<0.05) (Fig 5). With regard to control strains, the presence of *ecpA* was significantly associated with reduced biofilm biomass formation (median OD_492_: 0.220 vs 0.332, respectively for *ecpA*^+^ and *ecpA*^-^ strains; *p*<0.05) (Fig 6).

#### Biofilm formation and motility

Motility did not correlate with efficiency in biofilm biomass formation (S4 Fig). Particularly, APN24, RC13, RC14, CO8, and C11 strains showed neither swimming nor swarming motility, whereas they were able to form biofilm. No statistically significant differences were found among the motility patterns with respect to the biofilm amount formed.

#### Biofilm formation and antibiotic resistance

The mean amount of biofilm formed did not statistically differ either between resistant and susceptible strains, regardless of drug considered, or according to the MDR phenotype (S5 Fig). No differences were found in the prevalence of biofilm-producer strains between resistant and susceptible strains, regardless of the antibiotic considered (data not shown). However, a significantly higher percentage of moderate-producer strains were found in ampicillin-resistant strains, compared to susceptible ones (80 vs 20%, respectively; *p*<0.01).

## Discussion

In the present study we searched for a virulence repertoire distinctive for *E*. *coli* causing primitive APN. To this end, we examined the distribution of both genotypic (VFGs, phylogenetic groups), and phenotypic (antibiotic-resistance, biofilm formation, motility) traits among *E*. *coli* isolates from women with primitive APN, as compared to those isolated from women with RC and healthy controls.

### Clonal relatedness

The genome plasticity is the main driver for the phenotypic diversity and evolution of *E*. *coli* [14]. The high dynamicity of its genome is responsible for the relevant differences observed among pathogenic strains associated with human diseases. Pulsed field gel electrophoresis genomic typing we performed has demonstrated such high diversity showing an extremely heterogeneous population with a highly polyclonal distribution. The most genetically related strains, albeit not belonging to the same clone, were from healthy (CO15) and recurrent cystitis (RC8) groups. It is, therefore, plausible to infer that CO15 is a potential pathogenic strain that was residing innocuously within its reservoir. In this regard, previous studies [32, 33] showed that commensal *E*. *coli* may potentially serves as a source or reservoir of virulence genes for human pathogenesis.

### VFGs

A wide variety of virulence factors have been associated, epidemiologically or *in vivo*, with UPEC. Using PCR, we screened our entire collection of *E*. *coli* strains for 28 VFs, selected by their potential role described in the literature. Then, to visualize the distribution of VFGs and detect if specific combinations of VFGs were associated with APN we made a cluster analysis.

Overall, we found that *E*. *coli* causing APN or RC had a comparable mean aggregate VFG score, but significantly higher compared to apathogenic commensal strains (8.6 and 9.8 vs 6.2, respectively; p<0.05 and p<0.001, CO vs APN and RC, respectively). Particularly, APN and RC strains differed from apathogenic commensal ones in having a significantly higher prevalence of *fyuA* (yersiniabactin receptor for iron uptake), *usp*, and *malX* (pathogenicity-associated island marker) genes. The same virulence repertoire (*fyuA-usp-malX*) has been shown, in a mouse model, to be associated with killer strains [34].

Yersiniabactin has previously been associated with relapse and in vivo pathogenesis [35], whereas the uropathogenic-specific bacteriocin USP has been shown to be associated with pyelonephritis, prostatitis and bacteremia, and with enhanced virulence and fitness of pathogenic strains of *E*. *coli* [36–38].

Besides encoding virulence genes, pathogenicity islands may contribute to UPEC pathogenicity as well [39]. The presence of *malX*, coding for a pathogenicity-associated island marker of the pyelonephritis-associated CFT073 strain, was previously associated with persistence or relapse [18].

Among pathogenic strains, *iha* was significantly more prevalent in strains isolated from patients with APN, thus suggesting this adhesin, with its siderophore function, might play a relatively more important role than others examined (*papAH*, *papG I*, *papG II/III*, *sfa/focDE*, *focG*, *afa/draBC*, *bmaE*, *gafD*, *nfaE*, *sfaS*, and *ecpA* genes) in the pathogenesis of acute primitive pyelonephritis by *E*. *coli*.

On the contrary, multiple virulence factors were found to be apparently associated with the pathogenesis of RC since a higher prevalence of *iroN* (outer membrane receptor for catecholate siderophore salmochelin), *cnf1* (cytotoxic necrotizing factor 1), and *kpsMT-II* (group II-capsule, protecting against phagocytosis, opsonization and lysis)–was found in strains from patients with RC.

*iroN*, a novel catechole siderophore receptor that exhibits increased expression in urine, was found to be more prevalent among *E*. *coli* isolates from patients with UTI or bacteremia than among commensal strains, consistent with its being a virulence factor in extraintestinal infections.

*E*. *coli* is able to produce and inject toxins into the host uroepithelial cells, leading to different cell defense and cell damage events. Among these, CNF1 has been reported as being more prevalent in UPEC because it promotes bacterial attachment to and invasion of uroepithelial cells, both *in vitro* and *in vivo*, inducing cytoskeleton reorganization and modulation of inflammatory signaling pathways [14, 40].

The majority of the extraintestinal pathogenic *E*. *coli* strains, including UPEC, produce group II type capsules. Particularly, K2 capsule is essential for protection from complement-mediated killing in a murine model [17].

Cluster analysis performed by Principal Component Analysis indicated that *fyuA*+/*ecpA*+ (*E*. *coli* common pilus) and *iutA*+ (aerobactin receptor)/*iha*+ combinations were significantly associated respectively with uropathogenic strains (APN, RC) or APN, and conceivably could be considered as markers for predicting the pathogenic potential of a strain and, therefore, for innovative therapy strategies. No specific VFG combination was found in RC strains.

Interestingly, some VFGs were confined, although with low prevalence, to a single source group or to both uropathogenic groups. However, although these VFGs may contribute to the uropathogenesis in selected patients, they would not be ideal targets for anti-virulence interventions for the prevention of APN or RC. This is the case with *PapG I* (P fimbria adhesin—allele I), found in 2 out of 18 (11.1%) APN strains, and *rfc* (O4 lipopolysaccharide synthesis) and *cvaC* (colicin V) respectively observed only in 2 (10.5%) and 1 (5.25%) out of 19 RC strains. Although P fimbriae are associated with adherence to renal cells, our findings confirmed that their contribution to the pathogenesis of pyelonephritis remains equivocal [41].

*papAH* and *malX* were exclusive for uropathogenic strains having been found in APN and RC strains only, although with different frequencies. P fimbria-encoding *papAH* gene was present only in a minority of our uropathogenic strains, with a comparable prevalence in APN and RC strains (16.7 and 10.5%, respectively). In disagreement with these findings, previous studies showed *papAH* as being statistically associated with pyelonephritis rather than cystitis isolates [13, 20].

### Phylogroup prevalence and association with VFGs

*E*. *coli* strains can be assigned to one of the four well-recognized phylogenetic groups: A, B1, D1 and B2 [42]. Previous studies have evidenced that extraintestinal pathogenic *E*. *coli* strains belong mainly to phylogenetic groups B2, the most ancestral one, and D [15, 43]. Conversely, most of the APN and RC strains we tested in the present work belong to group B1, and to a lesser extent to phylogroups B2 and D (70.2% vs 5.4% and 8.1%, respectively; *p*<0.01). A point worth noting is the exclusive phylogenic arrangement observed for APN strains, that is the presence of phylogroup A and absence of phylogroup B2. The reasons for these discrepancies are unclear. They might be due to geographical variations [16, 44], reflect a study population with a greater number of clinical compromising conditions, or suggest that APN ad RC strains come from the commensal microbiota of the intestinal tract. Previous phylogenetic analyses have indeed reported that group B2 *E*. *coli* isolates are uncommon among commensal intestinal flora [25, 45]. Overall, these findings suggest that the phylogenetic background, as well as the VFG repertoire, is important for predicting the pathogenic potential in extraintestinal *E*. *coli*.

A link between phylogeny and virulence has been previously documented [15, 43]. Particularly, B2 and D strains causing urinary tract infections or newborn meningitis harbored a greater number of VFGs, as compared with strains from other phylogroups, and this number was proportional to its pathogenic potential [46]. Although we found no differences in VFG score among phylogroups within each study-group, only B1 strains showed the following virulence traits: namely, that *iha* is significantly associated with APN, *kpsMT-II* and *malX* are associated with RC, whereas *fyuA* is significantly more prevalent in clinical (APN and RC) than control strains.

The confinement of *focG* and *sfaS* to the B1 phylogroup may be due either to their recent arrival in this group or to barriers (whether from genetic or selection factors) to their horizontal movement into other groups.

To our knowledge, our results for the first time suggest that exploring VFs or other bacterial characteristics associated with phylogenetic B1 might be helpful in unveiling the pathogenesis behind APN and RC.

### Biofilm formation and its association with virulence factors

Biofilms are microbial communities consisting of bacterial cells adhering to each other and onto a biotic or abiotic substratum. They are clinically relevant since they are inherently resistant to antibiotics as well as phagocytosis and host defense mechanisms, thus allowing microorganisms to persist in the host.

In disagreement with previous works [37, 47], we found that the ability to adhere and form biofilm is highly conserved regardless of the source considered, with comparable prevalence rates (ranging from 83.3% to 73.6%, for APN and RC strains respectively). However, a great variation in the ability of pathogenic *E*. *coli* to form biofilms was observed, which confirmed previous studies [48, 49].

Although the capacity for biofilm formation was highly conserved, the efficiency in forming biofilm proved to be higher among uropathogenic strains in which the mean biofilm amount formed was significantly higher than with apathogenic strains. In support of this, the single strong biofilm producer strain belonged to APN and phylogroup A. These findings confirmed that biofilm production is one of the several putative virulence determinants possessed by UPEC causing APN or RC. In fact, even if not directly involved in invasiveness, higher amounts of biofilm formation may still result in an increased ability by strains to persist in the urinary tract, thus leading to recurrent UTIs.

In disagreement with previous studies [37, 47], no significant differences in biofilm formation were found among phylogroups.

Studying the mechanisms underlying biofilm formation may be of help in devising new therapeutic solutions to treat these infections. In this regaard, a variable association between biofilm formation and certain urovirulence genes in *E*. *coli* has been reported [35, 50–53]. In the present work, a clear trend towards a negative relationship between biofilm biomass formed and VFG aggregate score was observed in APN strains. This finding suggests that in *E*. *coli* a virulent strain may be able to decrease its virulence by forming a biofilm so that it can achieve persistent infection *in vivo*, a mechanism already described for other species [54, 55].

Considering the single VFGs tested, we found that the higher potential for biofilm formation by APN strains is positively associated with the presence of *iha* (iron-regulated gene homologue adhesion).

To the best of our knowledge, this is the first report suggesting that iron-regulated *iha*—codifying for an adhesin, siderophore receptor—may also have significant roles in biofilm formation, contrary to what was found by Kanamaru et al. [51].

In disagreement with Magistro et al. [53], we found that *iroN*, codifying for outer membrane receptor for catecholate siderophore salmochelin, hinders biofilm formation in UPEC. Similarly, the absence of *KpSMT*-*K1* (K1 capsule synthesis) significantly improves biofilm biomass levels, confirming the antibiofilm effect played by capsular polysaccharides by both shielding bacterial surface adhesins [50, 52] and releasing a soluble high-molecular-weight polysaccharide [52].

### Motility

Bacterial motility is thought to play an important role in virulence, correlating with the induction of various virulence determinants [56]. Flagella assist UPEC in the ascent from the bladder to the kidneys through the ureters, mediating bacterial adhesion and invasion, and reducing resistance to phagocytosis [57, 58]. In *E*. *coli*, two distinct types of flagellum have been implicated in different forms of motility [59]: i) conventional flagella, required for bacterial swimming in liquid medium; and ii) lateral flagella, required for bacterial swarming on surfaces or in viscous substances.

In the present study, we comparatively evaluated commensal and uropathogenic strains for motility patterns. All strains showed twitching motility, regardless of the study-group considered, whereas significant differences were found for both swarming and swimming motilities. Swarming motility is more prevalent in APN strains than commensal ones, thus confirming that during the early stages of infection bacterial pathogens are exposed to viscous environments, such as mucus overlaying urothelium, and may preferentially differentiate into swarmer cells to increase their growing area. Swarming has also been associated with pathogenesis, and enhanced resistance to both antibiotics and eukaryotic engulfment [60].

Interestingly, an opposite trend was observed for swimming motility whose prevalence was higher in commensal strains than those isolated from women with RC.

The flagellum controls many important functions other than motility, including biofilm formation. In *E*. *coli*, motility improves the chance of coming in contact with a surface, at which point type I pili are required to stabilize cell-to-surface attachment [61, 62]. Finally, motility facilitates the development of a mature biofilm by allowing movement along a surface, thereby helping spread the biofilm. However, our data do not support a direct role for motility in biofilm formation.

### Antibiotic resistance

Increasing resistance by pathogenic *E*. *coli* is a world-wide phenomenon that has appeared during the past three decades [63]. Previous studies revealed a high variability in the antibiotic-resistance rates of APN-causing *E*. *coli* strains, probably depending on the geographic regions and local or national antibiotic policy for treatment of UTI and other infections [3, 4, 64].

In the present study, no statistically significant differences in resistance rate were found among groups and phylogroups. Considering a cut-off point of 20% as the level of resistance at which an agent should no longer be used [65], our results clearly showed that ampicillin, amoxicillin/clavulanate, and cotrimoxazole should not be considered as an initial therapeutic regimen in patients with community-acquired APN or RC, without first obtaining antimicrobial susceptibility data. Conversely, the relatively low resistance rates to ciprofloxacin (11.2%) and gentamicin (5.6%) indicate that these antibiotics could be safely used as empirical agents. No resistance was found to carbapanems and nitrofurantoin.

It is worth noting that only one ESBL-producer strain belonged to the CO group. This finding, added to the comparable antibiotic resistance patterns observed both in UPEC and CO groups, clearly suggest that commensal isolates might represent a potential human health threat, as well as UPEC isolates.

No relationship was found between antibiotic-resistance and the efficiency in forming biofilm.

Association between VFGs and resistance is a complex phenomenon. Although previous investigations have shown that resistance to various classes of antimicrobial agent is associated with lack of uropathogenic virulence traits [20, 37, 66, 67], in this study no significant relationship was found, regardless of the antibiotic considered.

### Strengths and limitations of the present study

Strengths of the present study include: i) the extensive array of phenotypic and genotypic traits considered; ii) the analysis of their distribution by a multiple complementary analytical approach; iii) the use of prospectively collected strains for each study-group, isolated from the same place and time frame. However, this work also has several drawbacks: i) not having considered VFGs that could be specifically associated with APN or RC in women [68]; ii) lack of attention to VFG expression, since the presence of VFGs may be insufficient *per se* to define virulence; iii) biofilm formation *in vitro* might not predict biofilm formation *in vivo*; further work is warranted to evaluate whether the detection of biofilm production might be useful for selecting which patients may require short or prolonged antibiotic treatment; iv) the lack of clinical data for both RC and CO groups.

## Conclusions

In conclusion, our results indicate that, compared with commensal apathogenic isolates, *E*. *coli* from APN and RC show a significantly higher number of VFGs, thus indicating the virulence potential of UPEC strains.

However, in some cases commensal and UPEC isolates showed a comparable phylogenetic background and antibiotic-resistance pattern, and were genetically related. Commensal *E*. *coli* isolates might therefore have similar fitness properties to UPEC when adapting to an extraintestinal lifestyle, which, in turns, enable them to cause extraintestinal disease in humans, as does UPEC.

It is worth noting that some single VFGs were confined to a single pathogenic source group. Trying to identify syndrome-specific VFs that could be used as markers for detecting uropathogenic *E*. *coli*, we observed that *iha* may play a prevalent role in the pathogenesis of APN, as well as *iroN* and *KpsMT*-*II* in RC. Whether these VFs could act in concert with other known or unknown VFs in causing APN or RC, they could conceivably be used as targets for designing new prophylactic and/or therapeutic interventions [69].

Considering all the functional VFG groups screened, we did not find a specific pathogenic strategy that predisposes *E*. *coli* strains to cause persistence or relapse of UTI. On the contrary, the higher potential for biofilm formation we observed on the part of both APN and RC strains might offer a possible explanation in this regard. Lastly, we reported an exclusive phylogenic arrangement for APN strains, consisting in the presence of phylogroups A and B1, mostly associated with commensal strains, and the absence of phylogroup B2.

Further work is warranted to translate the knowledge gained in virulence and fitness mechanisms into developing therapeutics and vaccines.

## Supporting information

S1 Fig *In vitro* motility of *E*. *coli*. Swimming, swarming, and twitching motilities were measured using dedicated agars, as previously described [31]. Phylogenetic groups were evaluated using PCR triplex assay, according to Clermont et al. [9]: A, B1, B2, D, NT (non-typeable). Results are expressed as scatter plot, where bars indicate median value with interquartile range. Each dot is the average from two independent experiments with eight replicates of each strain per experiment (n = 16). No significant differences were found among groups by Kruskal-Wallis + Dunn’s multiple comparison post-test.
(DOCX)

S2 Fig Antibiotic-resistance and VFG score shown by *E*. *coli*. *E*. *coli* strains were isolated from patients with acute primitive pyelonephritis (APN), recurrent cystitis (RC) or from uninfected control subjects (CO). No significant relationship existed by Spearman’s correlation coefficient.
(DOCX)

S3 Fig Biofilm formation by E. coli strains from women with recurrent cystitis (RC), according to each virulence factor gene (VFG). The presence of VFGs was assessed by PCR. Biofilm forming ability onto polystyrene was spectrophotometrically assessed by crystal violet stain [29]. Each dot is the average from two independent experiments with eight replicates of each strain per experiment (n = 16). Horizontal bars are medians. No statistically significant differences were found by the Mann-Whitney test.
(DOCX)

S4 Fig Biofilm formation and motility. *E*. *coli* strains were isolated from patients with acute primitive pyelonephritis (APN), recurrent cystitis (RC) or from uninfected control subjects (CO). **A, C, E**) Correlation between motility and biofilm formation. Swimming (green squares), swarming (red triangles), and twitching (blue circles) motilities were performed as previously described [31]. Biofilm biomass was spectrophotometrically evaluated [29]. Spearman’s correlation coefficient indicated no significant (*p*<0.05) correlation. **B, D, F**) Biofilm formation stratified according motility patterns (swimming/swarming/twitching; + positivity,—negativity). Results are median values with interquartile ranges. No significant differences between groups were observed by Kruskal-Wallis + Dunn’s multiple comparison post-test (or Mann-Whitney test, for CO). Each dot is the average from two independent experiments with eight replicates of each strain per experiment (n = 16).
(DOCX)

S5 Fig Biofilm formation and antibiotic resistance. *E*. *coli* strains were isolated from patients with acute primitive pyelonephritis (APN), recurrent cystitis (RC) or from uninfected control subjects (CO). Antimicrobial susceptibility tests were performed by disk diffusion agar test and results interpreted according to EUCAST recommendations [26]. Strains were classified as “susceptible” (S) or “resistant” (R), whereas “Intermediate” strains were considered as “resistant”. A strain was defined as multidrug-resistant (MDR) if resistant to at least one agent in three or more antimicrobial categories [28]. Each dot is the average from two independent experiments with three replicates of each strain per experiment (n = 6). Results are median values with interquartile ranges. The Mann-Whitney test indicated no significant difference between groups.
(DOCX)

S1 Table Primers used in PCR assay to assess the presence of virulence factor genes (VFGs). *E*. *coli* strains were screened for the presence of 28 VFGs using PCR assay. See Materials and Methods for gene description.
(DOCX)

## Abbreviations

APN: acute pyelonephritis
RC: recurrent cystitis
CFU: colony-forming unit
PFGE: pulsed-field gel electrophoresis
VFGs: virulence factor genes
ESBL: extended spectrum beta-lactamase
MDR: multi-drug resistant
OD: optical density
UPEC: uropathogenic *E*. *coli*

## References

[1] Foxman B, Klemstine KL, Brown PD. Acute pyelonephritis in US hospitals in 1997: hospitalization and in-hospital mortality. Ann Epidemiol 2003;13(2): 144–150. 12559674 10.1016/s1047-2797(02)00272-7

[2] Tenner SM, Yadven MW, Kimmel PL. Acute pyelonephritis. Preventing complications through prompt diagnosis and proper therapy. Postgrad Med 1992;91(2): 261–268. 1738745 10.1080/00325481.1992.11701211

[3] Gupta K, Hooton TM, Wobbe CL, Stamm WE. The prevalence of antimicrobial resistance among uropathogens causing acute uncomplicated cystitis in young women. Int J Antimicrob Agents 1999;11: 305–308. 10394988 10.1016/s0924-8579(99)00035-7

[4] Czaja CA, Scholes D, Hooton TM, Stamm WE. Population-based epidemiologic analysis of acute pyelonephritis. Clin Infect Dis 2007;45(3): 273–280. doi: 10.1086/519268 17599303 10.1086/519268

[5] Terlizzi ME, Gribaudo G, Maffei ME. UroPathogenic *Escherichia coli* (UPEC) infections: virulence factors, bladder responses, antibiotic, and non-antibiotic antimicrobial strategies. Front Microbiol 2017;8: 1566 doi: 10.3389/fmicb.2017.01566 28861072 10.3389/fmicb.2017.01566 PMC5559502

[6] Hannan TJ, Totsika M, Mansfield KJ, Moore KH, Schembri MA, Hultgren SJ. Host–pathogen checkpoints and population bottlenecks in persistent and intracellular uropathogenic *Escherichia coli* bladder infection. FEMS Microbiol Rev 2012;36: 616–648. doi: 10.1111/j.1574-6976.2012.00339.x 22404313 10.1111/j.1574-6976.2012.00339.x PMC3675774

[7] Lane MC, Alteri CJ, Smith SN, Mobley HL. Expression of flagella is coincident with uropathogenic *Escherichia coli* ascension to the upper urinary tract. Proc Natl Acad Sci USA 2007;104: 16669–16674. doi: 10.1073/pnas.0607898104 17925449 10.1073/pnas.0607898104 PMC2034267

[8] Sarkar S, Ulett GC, Totsika M, Phan MD, Schembri MA. Role of capsule and O antigen in the virulence of uropathogenic *Escherichia coli*. PLoS One 2014;9: e94786 doi: 10.1371/journal.pone.0094786 24722484 10.1371/journal.pone.0094786 PMC3983267

[9] Clermont O, Bonacorsi S, Bingen E. Rapid and simple determination of the *Escherichia coli* phylogenetic group. Appl Environ Microbiol 2000;66: 4555–4558. 11010916 10.1128/aem.66.10.4555-4558.2000 PMC92342

[10] Gordon DM, Clermont O, Tolley H, Denamur E. Assigning *Escherichia coli* strains to phylogenetic groups: multi-locus sequence typing versus the PCR triplex method. Environ Microbiol 2008;10: 2484–2496. doi: 10.1111/j.1462-2920.2008.01669.x 18518895 10.1111/j.1462-2920.2008.01669.x

[11] Johnson JR, Stell AL. Extended virulence genotypes of *Escherichia coli* strains from patients with urosepsis in relation to phylogeny and host compromise. J Infect Dis 2000;181: 261–272. doi: 10.1086/315217 10608775 10.1086/315217

[12] Johnson JR, Russo TA, Tarr PI, Carlino U, Bilge SS, Vary JC Jr, et al Molecular epidemiological and phylogenetic associations of two novel putative virulence genes, *iha* and *iroN*(*E. coli*), among *Escherichia coli* isolates from patients with urosepsis. Infect Immun 2000;68(5): 3040–3047. 10769012 10.1128/iai.68.5.3040-3047.2000 PMC97527

[13] Johnson JR, Kuskowski MA, Gajewski A, Soto S, Horcajada JP, Jimenez de Anta MT, et al Extended virulence genotypes and phylogenetic background of *Escherichia coli* isolates from patients with cystitis, pyelonephritis or prostatitis. J Infect Dis 2005;191: 46e50. 15593002 10.1086/426450

[14] Johnson JR, Owens K, Gajewski A, Kuskowski MA. Bacterial characteristics in relation to clinical source of *Escherichia coli* isolates from women with acute cystitis or pyelonephritis and uninfected women. J Clin Microbiol 2005;43: 6064–6072. doi: 10.1128/JCM.43.12.6064-6072.2005 16333100 10.1128/JCM.43.12.6064-6072.2005 PMC1317206

[15] Escobar-Páramo P, Clermont O, Blanc-Potard AB, Bui H, Le Bouguénec C, Denamur E. A specific genetic background is required for acquisition and expression of virulence factors in *Escherichia coli*. Mol Biol Evol 2004;21(6): 1085–1094. doi: 10.1093/molbev/msh118 15014151 10.1093/molbev/msh118

[16] Grude N, Potaturkina-Nesterova NI, Jenkins A, Strand L, Nowrouzian FL, Nyhus J, et al A comparison of phylogenetic group, virulence factors and antibiotic resistance in Russian and Norwegian isolates of *Escherichia coli* from urinary tract infection. Clin Microbiol Infect 2007;13(2): 208–211. doi: 10.1111/j.1469-0691.2006.01584.x 17328737 10.1111/j.1469-0691.2006.01584.x

[17] Buckles EL, Wang X, Lane MC, Lockatell CV, Johnson DE, Rasko DA, et al Role of the K2 capsule in *Escherichia coli* urinary tract infection and serum resistance. J Infect Dis 2009;199: 1689–1697. doi: 10.1086/598524 19432551 10.1086/598524 PMC3872369

[18] Ejrnæs K, Stegger M, Reisner A, Ferry S, Monsen T, Holm SE, et al Characteristics of *Escherichia coli* causing persistence or relapse of urinary tract infections: phylogenetic groups, virulence factors and biofilm formation. Virulence 2011;2(6): 528–537. doi: 10.4161/viru.2.6.18189 22030858 10.4161/viru.2.6.18189

[19] Kudinha T, Kong F, Johnson JR, Andrew SD, Anderson P, Gilbert GL. Multiplex PCR-based reverse line blot assay for simultaneous detection of 22 virulence genes in uropathogenic *Escherichia coli*. Appl Environ Microbiol 2012;78(4): 1198–1202. doi: 10.1128/AEM.06921-11 22156422 10.1128/AEM.06921-11 PMC3272995

[20] Er DK, Dundar D, Uzuner H, Osmani A. Relationship between phylogenetic groups, antibiotic resistance and patient characteristics in terms of adhesin genes in cystitis and pyelonephritis isolates of *Escherichia coli*. Microb Pathog 2015;89: 188–194. doi: 10.1016/j.micpath.2015.10.014 26518125 10.1016/j.micpath.2015.10.014

[21] Delcaru C, Podgoreanu P, Alexandru I, Popescu N, Măruţescu L, Bleotu C, et al Antibiotic resistance and virulence phenotypes of recent bacterial strains isolated from urinary tract infections in elderly patients with prostatic disease. Pathogens 2017;6(2): E22 doi: 10.3390/pathogens6020022 28561794 10.3390/pathogens6020022 PMC5488656

[22] Horan TC, Andrus M, Dudeck MA. CDC/NHSN surveillance definition of health care-associated infection and criteria for specific types of infections in the acute care setting. Am J Infect Control 2008;36: 309–332. doi: 10.1016/j.ajic.2008.03.002 18538699 10.1016/j.ajic.2008.03.002

[23] Tenover FC, Arbeit RD, Goering RV, Mickelsen PA, Murray BE, Persing DH, et al Interpreting chromosomal DNA restriction patterns produced by pulsed-field gel electrophoresis: criteria for bacterial strain typing. J Clin Microbiol 1995;33: 2233–2239. 7494007 10.1128/jcm.33.9.2233-2239.1995 PMC228385

[24] Blackburn D, Husband A, Saldaña Z, Nada RA, Klena J, Qadri F, et al Distribution of the *Escherichia coli* common pilus among diverse strains of human enterotoxigenic *E*. *coli*. J Clin Microbiol 2009;47(6): 1781–1784. doi: 10.1128/JCM.00260-09 19357209 10.1128/JCM.00260-09 PMC2691072

[25] Kudinha T, Johnson JR, Andrew SD, Kong F, Anderson P, Gilbert GL. Distribution of phylogenetic groups, sequence type ST131, and virulence-associated traits among *Escherichia coli* isolates from men with pyelonephritis or cystitis and healthy controls. Clin Microbiol Infect 2013;19(4): E173–180. doi: 10.1111/1469-0691.12123 23398521 10.1111/1469-0691.12123

[26] The European Committee on Antibiotic Susceptibility Testing (EUCAST): Breakpoint Table for interpretation of MICs and zone diameters. Version 7.1, 2017. http://www.eucast.org.

[27] Robin F, Delmas J, Schweitzer C, Bonnet R. Evaluation of the Vitek-2 extended-spectrum beta-lactamase test against non-duplicate strains of *Enterobacteriaceae* producing a broad diversity of well-characterised beta-lactamases. Clin Microbiol Infect 2008;14: 148–154. doi: 10.1111/j.1469-0691.2007.01893.x 18076663 10.1111/j.1469-0691.2007.01893.x

[28] Magiorakos AP, Srinivasan A, Carey RB, Carmeli Y, Falagas ME, Giske CG, et al Multidrug-resistant, extensively drug-resistant and pandrug-resistant bacteria: an international expert proposal for interim standard definitions for acquired resistance. Clin Microbiol Infect 2012;18(3): 268–281. doi: 10.1111/j.1469-0691.2011.03570.x 21793988 10.1111/j.1469-0691.2011.03570.x

[29] Pompilio A, Pomponio S, Crocetta V, Gherardi G, Verginelli F, Fiscarelli E, et al Phenotypic and genotypic characterization of *Stenotrophomonas maltophilia* isolates from patients with cystic fibrosis: genome diversity, biofilm formation, and virulence. BMC Microbiol 2011;5(11): 159. 10.1186/1471-2180-11-159 PMC3146419 21729271

[30] Stepanović S, Vuković D, Hola V, Di Bonaventura G, Djukić S, Cirković I, et al Quantification of biofilm in microtiter plates: overview of testing conditions and practical recommendations for assessment of biofilm production by staphylococci. APMIS 2007;115(8): 891–899. doi: 10.1111/j.1600-0463.2007.apm_630.x 17696944 10.1111/j.1600-0463.2007.apm_630.x

[31] Pompilio A, Piccolomini R, Picciani C, D'Antonio D, Savini V, Di Bonaventura G. Factors associated with adherence to and biofilm formation on polystyrene by *Stenotrophomonas maltophilia*: the role of cell surface hydrophobicity and motility. FEMS Microbiol Lett 2008;287(1): 41–47. doi: 10.1111/j.1574-6968.2008.01292.x 18681866 10.1111/j.1574-6968.2008.01292.x

[32] Burman WJ, Breese PE, Murray BE, Singh KY, Batal HA, Mackenzie TD, et al Conventional and molecular epidemiology of trimethoprim-sulfamethoxazole resistance among urinary *Escherichia coli* isolates. Am J Med 2003;115: 358–364. 14553870 10.1016/s0002-9343(03)00372-3

[33] Moulin-Schouleur M, Schouler C, Tailliez P, Kao MR, Bree A, Germon P, et al Common virulence factors and genetic relationships between O18: K1: H7 *Escherichia coli* isolates of human and avian origin. J Clin Microbiol 2006;44: 3484–3492. doi: 10.1128/JCM.00548-06 17021071 10.1128/JCM.00548-06 PMC1594794

[34] Johnson JR, Clermont O, Menard M, Kuskowski MA, Picard B, Denamur E. Experimental mouse lethality of *Escherichia coli* isolates, in relation to accessory traits, phylogenetic group and ecological source. J Infect Dis 2006;194: 1141–1150. doi: 10.1086/507305 16991090 10.1086/507305

[35] Soto SM, Smithson A, Horcajada JP, Martinez JA, Mensa JP, Vila J. Implication of biofilm formation in the persistence of urinary tract infection caused by uropathogenic *Escherichia coli*. Clin Microbiol Infect 2006;12: 1034–1036. doi: 10.1111/j.1469-0691.2006.01543.x 16961644 10.1111/j.1469-0691.2006.01543.x

[36] Yamamoto S, Nakano M, Terai A, Yuri K, Nakata K, Nair GB, et al The presence of the virulence island containing the usp gene in uropathogenic *Escherichia coli* is associated with urinary tract infection in an experimental mouse model. J Urol 2001;165: 1347–1351. 11257714

[37] Rijavec M, Müller-Premru M, Zakotnik B, Zgur-Bertok D. Virulence factors and biofilm production among *Escherichia coli* strains causing bacteraemia of urinary tract origin. J Med Microbiol 2008;57(Pt 11): 1329–1334. doi: 10.1099/jmm.0.2008/002543-0 18927408 10.1099/jmm.0.2008/002543-0

[38] Ostblom A, Adlerberth I, Wold AE, Nowrouzian FL. Pathogenicity island markers, virulence determinants *malx* and *usp*, and the capacity of *Escherichia coli* to persist in infants’ commensal microbiotas. Appl Environ Microbiol 2011;77(7): 2303–2308. doi: 10.1128/AEM.02405-10 21317254 10.1128/AEM.02405-10 PMC3067437

[39] Lloyd AL, Henderson TA, Vigil PD, Mobley HL. Genomic islands of uropathogenic *Escherichia coli* contribute to virulence. J Bacteriol 2009;191(11): 3469–3481. doi: 10.1128/JB.01717-08 19329634 10.1128/JB.01717-08 PMC2681901

[40] Hagberg L, Jodal U, Korhonen TK, Lidin-Janson G, Lindberg U, Svanborg EC. Adhesion, hemagglutination and virulence of *Escherichia coli* causing urinary tract infections. Infect Immun 1981;31: 564–570. 7012012 10.1128/iai.31.2.564-570.1981 PMC351345

[41] Subashchandrabose S, Mobley HL. Virulence and fitness determinants of uropathogenic *Escherichia coli*. Microbiol Spectr 2015;3(4). doi: 10.1128/microbiolspec.UTI-0015-2012 26350328 10.1128/microbiolspec.UTI-0015-2012 PMC4566162

[42] Herzer PJ, Inouye S, Inouye M, Whittam TS. Phylogenetic distribution of branched RNA-linked multi-copy single-stranded DNA among natural isolates of *Escherichia coli*. J Bacteriol 1990;172: 6175–6181. 1699928 10.1128/jb.172.11.6175-6181.1990 PMC526797

[43] Johnson JR, Kuskowski MA, O’Bryan TT, Maslow JN. Epidemiological correlates of virulence genotype and phylogenetic background among *Escherichia coli* blood isolates from adults with diverse-source bacteremia. J Infect Dis 2002;185: 1439–1447. doi: 10.1086/340506 11992279 10.1086/340506

[44] Martìnez JA, Soto S, Fabrega A, Almela M, Mensa J, Soriano A, et al Relationship of phylogenetic background, biofilm production, and time to detection of growth in blood culture vials with clinical variables and prognosis associated with *Escherichia coli* bacteremia. J Clin Microbiol 2006;44: 1468–1474. doi: 10.1128/JCM.44.4.1468-1474.2006 16597878 10.1128/JCM.44.4.1468-1474.2006 PMC1448679

[45] Navidinia M, Peerayeh SN, Fallah F, Bakhshi B, Sajadinia RS. Phylogenetic grouping and pathotypic comparison of urine and fecal *Escherichia coli* isolates from children with urinary tract infection. Braz J Microbiol 2014;45(2): 509–514. 25242935 10.1590/s1517-83822014000200019 PMC4166276

[46] Picard B, Garcia JS, Gouriou S, Duriez P, Brahimi N, Bingen E, et al 1999. The link between phylogeny and virulence in *Escherichia coli* extraintestinal infection. Infect Immun 1999;67: 546–553. 9916057 10.1128/iai.67.2.546-553.1999 PMC96353

[47] Soto SM, Smithson A, Martinez JA, Horcajada JP, Mensa J, Vila J. Biofilm formation in uropathogenic *Escherichia coli* strains: relationship with prostatitis, urovirulence factors and antimicrobial resistance. J Urol 2007;177: 365–368. doi: 10.1016/j.juro.2006.08.081 17162092 10.1016/j.juro.2006.08.081

[48] Pratt LA, Kolter R. Genetic analysis of *Escherichia coli* biofilm formation: roles of flagella, motility, chemotaxis and type I pili. Mol Microbiol 1998;30: 285–293. 9791174 10.1046/j.1365-2958.1998.01061.x

[49] Reisner A, Krogfelt KA, Klein BM, Zechner EL, Molin S. *In vivo* biofilm formation of commensal and pathogenic *Escherichia coli* strains: impact of environmental and genetic factors. J Bacteriol 2006;188: 3572–3581. doi: 10.1128/JB.188.10.3572-3581.2006 16672611 10.1128/JB.188.10.3572-3581.2006 PMC1482849

[50] Schembri MA, Dalsgaard D, Klemm P. Capsule shields the function of short bacterial adhesins. J Bacteriol 2004;186: 1249–1257. doi: 10.1128/JB.186.5.1249-1257.2004 14973035 10.1128/JB.186.5.1249-1257.2004 PMC344426

[51] Kanamaru S, Kurazono H, Terai A, Monden K, Kumon H, Mizunoe Y, et al Increased biofilm formation in *Escherichia coli* isolated from acute prostatitis. Int J Antimicrob Agents 2006;28(Suppl 1): S21–S25. 16828264 10.1016/j.ijantimicag.2006.05.006

[52] Valle J, Da Re S, Henry N, Fontaine T, Balestrino D, Latour-Lambert P, et al Broad-spectrum biofilm inhibition by a secreted bacterial polysaccharide. Proc Natl Acad Sci USA 2006;33: 12558–12563. 10.1073/pnas.0605399103 PMC1567917 16894146

[53] Magistro G, Hoffmann C, Schubert S. The salmochelin receptor IroN itself, but not salmochelin-mediated iron uptake promotes biofilm formation in extraintestinal pathogenic *Escherichia coli* (ExPEC). Int J Med Microbiol 2015;305(4–5): 435–445. doi: 10.1016/j.ijmm.2015.03.008 25921426 10.1016/j.ijmm.2015.03.008

[54] Li L, Zhou R, Li T, Kang M, Wan Y, Xu Z, et al Enhanced biofilm formation and reduced virulence of *Actinobacillus pleuropneumoniae luxS* mutant. Microb Pathog 2008;45(3): 192–200. doi: 10.1016/j.micpath.2008.05.008 18585450 10.1016/j.micpath.2008.05.008

[55] Wang Y, Zhang W, Wu Z, Lu C. Reduced virulence is an important characteristic of biofilm infection of *Streptococcus suis*. FEMS Microbiol Lett 2011;316(1): 36–43. doi: 10.1111/j.1574-6968.2010.02189.x 21204925 10.1111/j.1574-6968.2010.02189.x

[56] Partridge JD, Harshey RM. Swarming: flexible roaming plans. J Bacteriol 2013;195: 909–918. doi: 10.1128/JB.02063-12 23264580 10.1128/JB.02063-12 PMC3571328

[57] Zhou M, Yang Y, Chen P, Hu H, Hardwidge PR, Zhu G. More than a locomotive organelle: flagella in *Escherichia coli*. Appl Microbiol Biotechnol 2015;99(21): 8883–8890. doi: 10.1007/s00253-015-6946-x 26346269 10.1007/s00253-015-6946-x

[58] Liu F, Fu J, Liu C, Chen J, Sun M, Chen H, et al Characterization and distinction of two flagellar systems in extraintestinal pathogenic *Escherichia coli* PCN033. Microbiol Res 2017;196: 69–79. doi: 10.1016/j.micres.2016.11.013 28164791 10.1016/j.micres.2016.11.013

[59] Swiecicki JM, Sliusarenko O, Weibel DB. From swimming to swarming: *Escherichia coli* cell motility in two-dimensions. Integr Biol (Camb) 2013;5(12): 1490–1494. 24145500 10.1039/c3ib40130h PMC4222179

[60] Butler MT, Wang Q, Harshey RM. Cell density and mobility protect swarming bacteria against antibiotics. Proc Natl Acad Sci USA 2010;107:3776–3781. doi: 10.1073/pnas.0910934107 20133590 10.1073/pnas.0910934107 PMC2840483

[61] Prüss BM, Besemann C, Denton A, Wolfe AJ. A complex transcription network controls the early stages of biofilm development by *Escherichia coli*. J Bacteriol 2006;188(11): 3731–3739. doi: 10.1128/JB.01780-05 16707665 10.1128/JB.01780-05 PMC1482888

[62] Ong CLY, Ulett GC, Mabbett AN, Beaston SA, Webb RI, Monaghan W, et al Identification of type 3 fimbriae in uropathogenic *Escherichia coli* reveals a role in biofilm formation. J Bacteriol 2008;190: 1054–1063. doi: 10.1128/JB.01523-07 18055599 10.1128/JB.01523-07 PMC2223576

[63] Talan DA, Krishnadasan A, Abrahamian FM, Stamm WE, Moran GJ; EMERGEncy ID NET Study Group. Prevalence and risk factor analysis of trimethoprim-sulfamethoxazole- and fluoroquinolone-resistant *Escherichia coli* infection among emergency department patients with pyelonephritis. Clin Infect Dis 2008;47: 1150–1158. doi: 10.1086/592250 18808361 10.1086/592250

[64] Jung YS, Shin HS, Rim H. The influence of chronic renal failure on the spectrum and antimicrobial susceptibility of uropathogens in community-acquired acute pyelonephritis presenting as a positive urine culture. BMC Infect Dis 2011;11: 102 doi: 10.1186/1471-2334-11-102 21507269 10.1186/1471-2334-11-102 PMC3095993

[65] Gupta K, Hooton TM, Stamm WE. Increasing antimicrobial resistance and the management of uncomplicated community-acquired urinary tract infections. Ann Intern Med 2001;135: 41–50. 11434731 10.7326/0003-4819-135-1-200107030-00012

[66] Horcajada JP, Soto S, Gajewski A, Smithson A, Jimènez de Anta MT, Mensa J, et al Quinolone-resistant uropathogenic *Escherichia coli* strains from phylogenetic group B2 have fewer virulence factors than their susceptible counterparts. J Clin Microbiol 2005;43: 2962–2964. doi: 10.1128/JCM.43.6.2962-2964.2005 15956432 10.1128/JCM.43.6.2962-2964.2005 PMC1151912

[67] Moreno E, Planells I, Prats AM, Moreno G, Andreu A. Comparative study of *Escherichia coli* virulence determinants in strains causing urinary tract bacteremia versus strains causing pyelonephritis and other sources of bacteremia. Diagn Microbiol Infect Dis 2005;53: 93–99. doi: 10.1016/j.diagmicrobio.2005.05.015 16168618 10.1016/j.diagmicrobio.2005.05.015

[68] Welch R, Burland V, Plunkett G III, Redford P, Roesch P, Rasko D, et al Extensive mosaic structure revealed by the complete genome sequence of uropathogenic *Escherichia coli*. Proc Natl Acad Sci USA 2002;99: 17020–17024. doi: 10.1073/pnas.252529799 12471157 10.1073/pnas.252529799 PMC139262

[69] Alteri CJ, Hagan EC, Sivick KE, Smith SN, Mobley HL. Mucosal immunization with iron receptor antigens protects against urinary tract infection. PLoS Pathog 2009;5: e1000586 doi: 10.1371/journal.ppat.1000586 19806177 10.1371/journal.ppat.1000586 PMC2736566

