# Supplementary material for: Phylogenetic relationships, biofilm formation, motility, antibiotic resistance and extended virulence genotypes among Escherichia coli strains from women with community-onset primitive acute pyelonephritis
Source: PLoS One. 2018 May 14;13(5):e0196260. doi: 10.1371/journal.pone.0196260 (PMC5951556; doi:10.1371/journal.pone.0196260)
Supplement: S1 Table — E. coli strains were screened for the presence of 28 VFGs using PCR assay. See Materials and Methods for gene description. (DOCX) [file pone.0196260.s006.docx]

| **VFG**  **[reference]** | **GenBank accession n.** | **F-primer (5’-3’)** | **R-primer (5’-3’)** | **Product (bp)** |
| --- | --- | --- | --- | --- |
| *papAH*  [11] | X61239 | atggcagtggtgtcttttggtg | cgtcccaccatacgtgctcttc | 717 |
| *papG* - allele II/III  [11] | X61238 | ctgtaattacggaagtgatttctg | actatccggctccggataaaccat | 1070 |
| *papG -* allele I  [11] | X61239 | CTGTAATTACGGAAGTGATTTCTG | tccagaaatagctcatgtaacccg | 1190 |
| *sfa/focDE*  [11] | unpublished | ctccggagaactgggtgcatcttac | cggaggagtaattacaaacctggca | 410 |
| *focG*  [11] | S68237 | cagcacaggcagtggatacga | gaatgtcgcctgcccattgct | 364 |
| *iha*  [12] | AF126104 | CTGGCGGAGGCTCTGAGATCA | TCCTTAAGCTCCCGCGGCTGA | 827 |
| *afa*/*draBC*  [11] | X76688 | ggcagagggccggcaacaggc | cccgtaacgcgccagcatctc | 594 |
| *bmaE*  [11] | M15677 | atggcgctaacttgccatgctg | agggggacatatagcccccttc | 507 |
| *gafD*  [11] | L33969 | tgttggaccgtctcagggctc | ctcccggaactcgctgttact | 952 |
| *nfaE*  [11] | S61970 | gcttactgattctgggatgga | cggtggccgagtcatatgcca | 559 |
| *sfaS*  [11] | S53210 | gtggatacgacgattactgtg | ccgccagcattccctgtattc | 244 |
| *ecpA*  [24] |  | TGAAAAAAAAGGTTCTGGCAATAGC | CGCTGATGAGGAGAAAGTGAA |  |
| *iutA*  [11] | X05874 | ggctggacatcatgggaactgg | cgtcgggaacgggtagaatcg | 302 |
| *fyuA*  [11] | Z38064 | tgattaaccccgcgacgggaa | cgcagtaggcacgatgttgta | 787 |
| *iroNE.coli*  [12] | AF135597 | AAGTCAAAGCAGGGGTTGCCCG | GACGCCGACATTAAGACGCAG | 665 |
| *hlyA*  [11] | M10133 | aacaaggataagcactgttctggct | accatataagcggtcattcccgtca | 1177 |
| *cnf1*  [11] | X70670 | aagatggagtttcctatgcaggag | cattcagagtcctgccctcattatt | 498 |
| *cdtB*  [11] | U04208 | AAATCACCAAGAATCATCCAGTTA | AAATCTCCTGCAATCAATCCAGTTTA | 430 |
| *kpsMT*-II  [11] | X53819 | gcgcatttgctgatactgttg | catccagacgataagcatgagca | 272 |
| *kpsMT*-III  [11] | AF007777 | tcctcttgctactattccccct | aggcgtatccatccctcctaac | 392 |
| *kpsMT-K1*  [11] |  | tagcaaacgttctattggtgc | catccagacgataagcatgagca | 153 |
| *rfc*  [11] | U39042 | atccatcaggaggggactgga | aaccataccaaccaatgcgag | 788 |
| *cvaC*  [11] | X57525 | cacacacaaacgggagctgtt | cttcccgcagcatagttccat | 680 |
| *traT*  [11] | J01769 | ggtgtggtgcgatgagcacag | cacggttcagccatccctgag | 290 |
| *ibeA*  [11] | L42624 | aggcaggtgtgcgccgcgtac | tggtgctccggcaaaccatgc | 171 |
| *usp*  [19] | AB056434 | CTTCACCCGTATGAACACCAT | GCTGCCTGGTGTTGTAACAG | 448 |
| *ompT*  [19] | X06903 | GTCGAGTTGACTGACTTTTCG | TGGTGGAATCATCTGATAAC | 496 |
| *malX*  [11] | AF003742 | ggacatcctgttacagcgcgca | tcgccaccaatcacagccgaac | 930 |
