# Supplementary material for: Phylogenetic relationships, biofilm formation, motility, antibiotic resistance and extended virulence genotypes among Escherichia coli strains from women with community-onset primitive acute pyelonephritis
Source: PLoS One. 2018 May 14;13(5):e0196260. doi: 10.1371/journal.pone.0196260 (PMC5951556; doi:10.1371/journal.pone.0196260)
Supplement: Supplementary file 2 [file pone.0196260.s001.docx]

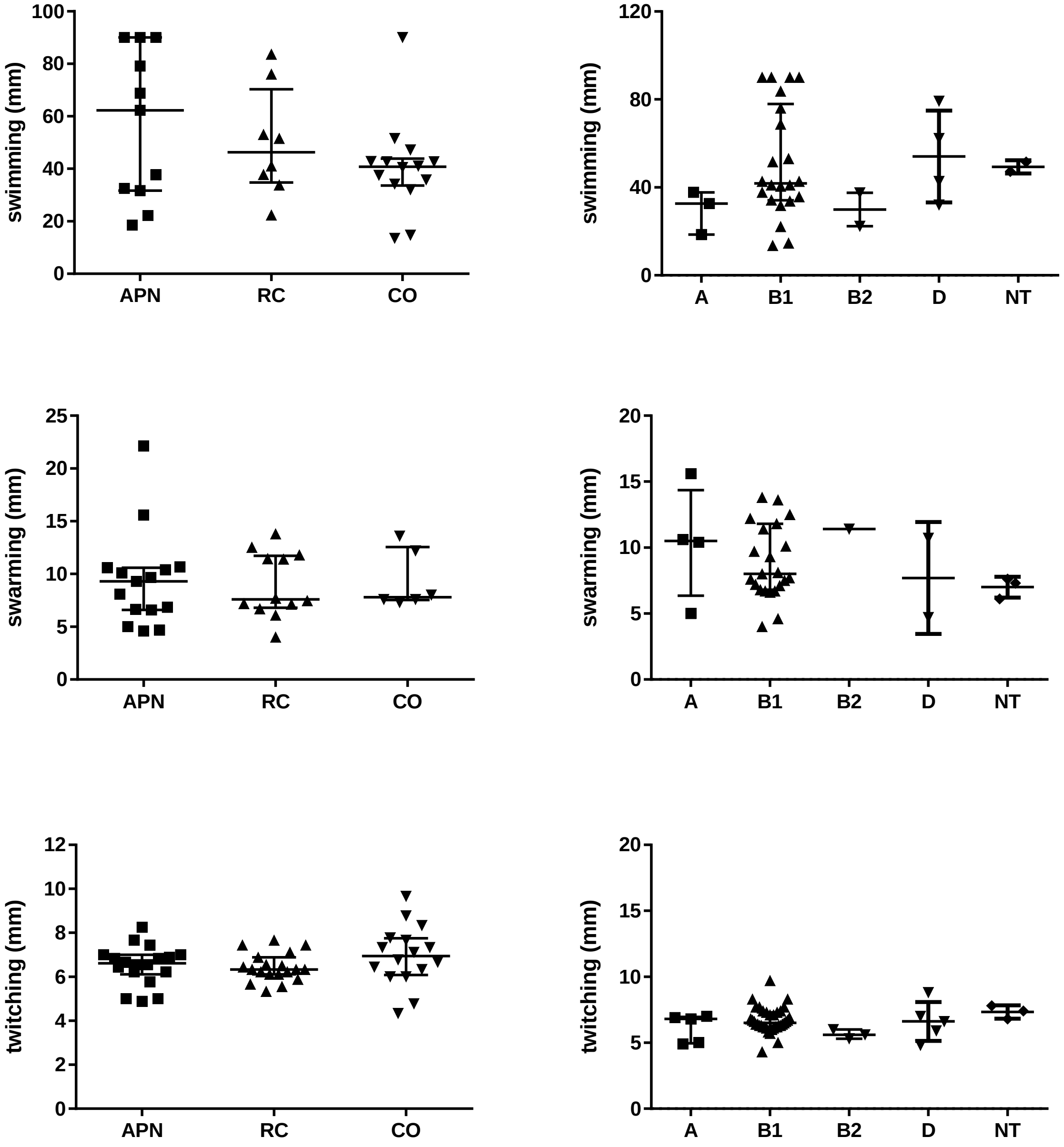

swimming (mm)
0
20
40
60
80
100
APN
RC
CO
swimming (mm)
0
40
80
120
A
B1
B2
D
NT
swarming (mm)
0
5
10
15
20
25
APN
RC
CO
swarming (mm)
0
5
10
15
20
A
B1
B2
D
NT
twitching (mm)
0
2
4
6
8
10
12
APN
RC
CO
twitching (mm)
0
5
10
15
20
A
B1
B2
D
NT
