# Supplementary material for: Phylogenetic relationships, biofilm formation, motility, antibiotic resistance and extended virulence genotypes among Escherichia coli strains from women with community-onset primitive acute pyelonephritis
Source: PLoS One. 2018 May 14;13(5):e0196260. doi: 10.1371/journal.pone.0196260 (PMC5951556; doi:10.1371/journal.pone.0196260)
Supplement: Supplementary file 3 [file pone.0196260.s002.docx]

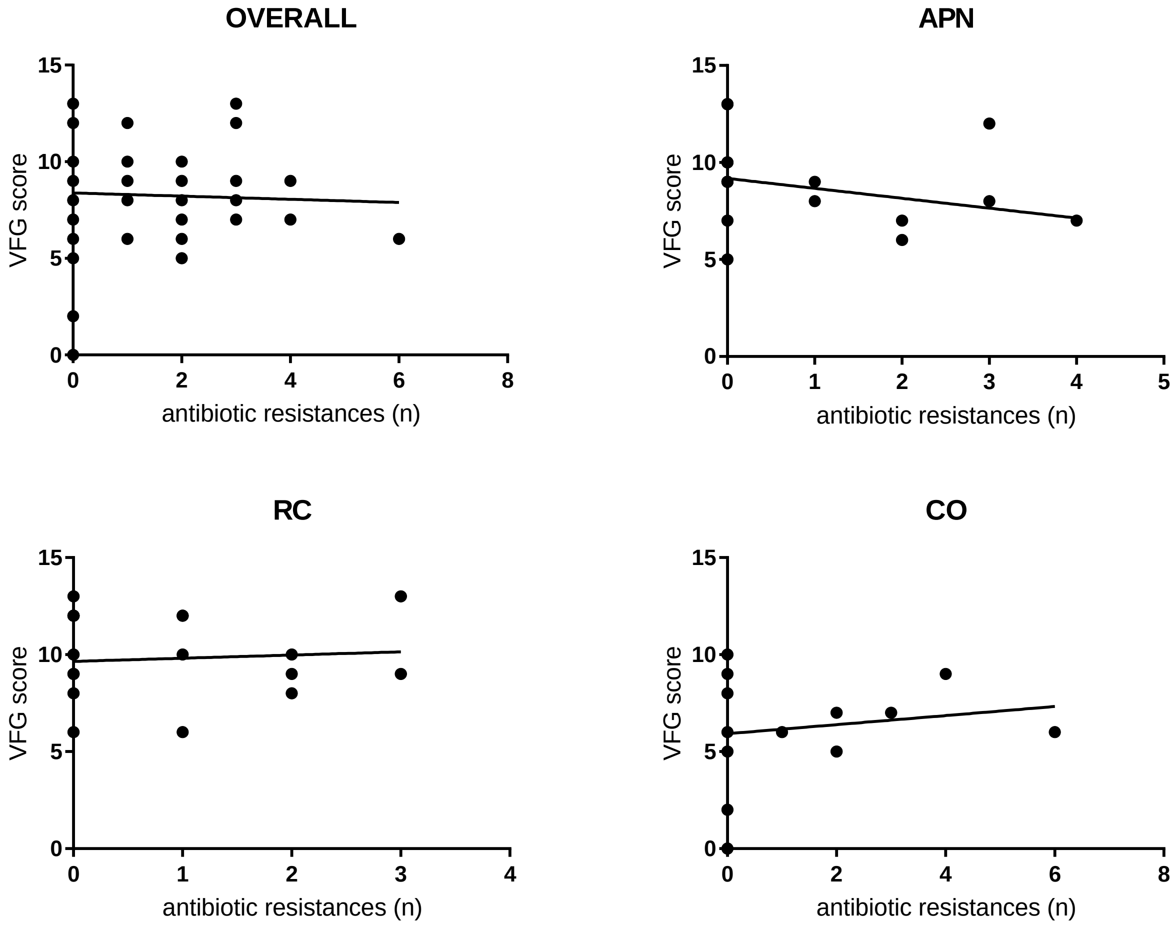
OVERALL
VFG score
antibiotic resistances (n)
APN
VFG score
antibiotic resistances (n)
RC
VFG score
antibiotic resistances (n)
CO
VFG score
antibiotic resistances (n)
