# Supplementary material for: Phylogenetic relationships, biofilm formation, motility, antibiotic resistance and extended virulence genotypes among Escherichia coli strains from women with community-onset primitive acute pyelonephritis
Source: PLoS One. 2018 May 14;13(5):e0196260. doi: 10.1371/journal.pone.0196260 (PMC5951556; doi:10.1371/journal.pone.0196260)
Supplement: Supplementary file 4 [file pone.0196260.s003.docx]

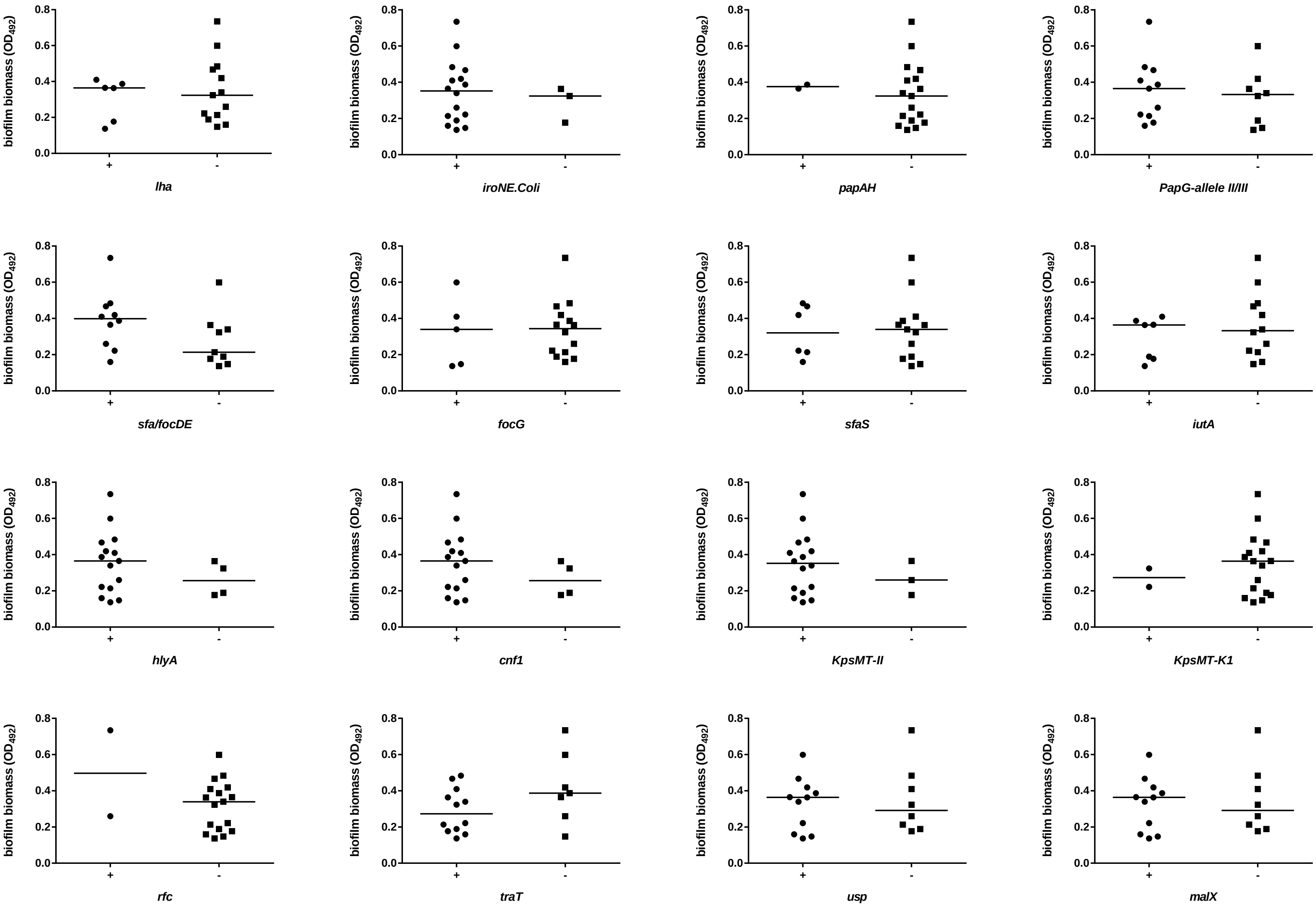
biofilm biomass (OD_{492})
0.0
0.2
0.4
0.6
0.8
+
-
lha
iroNE.Coli
papAH
PapG-allele II/III
sfa/focDE
focG
sfaS
iutA
hlyA
cnf1
KpsMT-II
KpsMT-K1
rfc
traT
usp
malX
