# Supplementary material for: Phylogenetic relationships, biofilm formation, motility, antibiotic resistance and extended virulence genotypes among Escherichia coli strains from women with community-onset primitive acute pyelonephritis
Source: PLoS One. 2018 May 14;13(5):e0196260. doi: 10.1371/journal.pone.0196260 (PMC5951556; doi:10.1371/journal.pone.0196260)
Supplement: Supplementary file 5 [file pone.0196260.s004.docx]

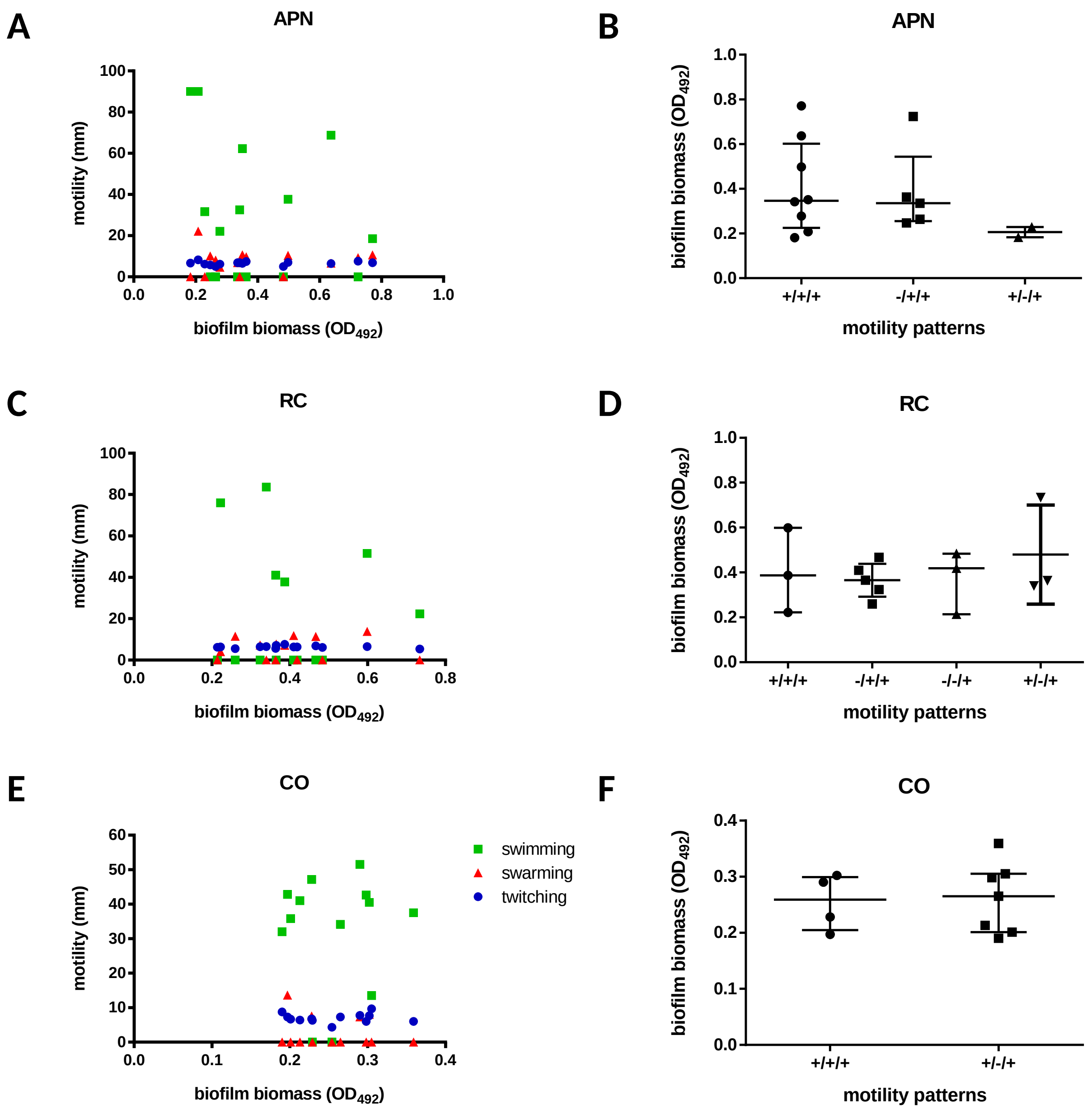
A
APN
motility (mm)
100
80
60
40
20
0
0.0
0.2
0.4
0.6
0.8
1.0
biofilm biomass ($OD_{492}$)
B
APN
biofilm biomass ($OD_{492}$)
1.0
0.8
0.6
0.4
0.2
0.0
+/+/+
-/+/+
+/-/+
motility patterns
C
RC
motility (mm)
100
80
60
40
20
0
0.0
0.2
0.4
0.6
0.8
biofilm biomass ($OD_{492}$)
D
RC
biofilm biomass ($OD_{492}$)
1.0
0.8
0.6
0.4
0.2
0.0
+/+/+
-/+/+
-/-/+
+/-/+
motility patterns
E
CO
motility (mm)
60
50
40
30
20
10
0
0.0
0.1
0.2
0.3
0.4
biofilm biomass ($OD_{492}$)
swimming
swarming
twitching
F
CO
biofilm biomass ($OD_{492}$)
0.4
0.3
0.2
0.1
0.0
+/+/+
+/-/+
motility patterns
