# Supplementary material for: Phylogenetic relationships, biofilm formation, motility, antibiotic resistance and extended virulence genotypes among Escherichia coli strains from women with community-onset primitive acute pyelonephritis
Source: PLoS One. 2018 May 14;13(5):e0196260. doi: 10.1371/journal.pone.0196260 (PMC5951556; doi:10.1371/journal.pone.0196260)
Supplement: Supplementary file 6 [file pone.0196260.s005.docx]

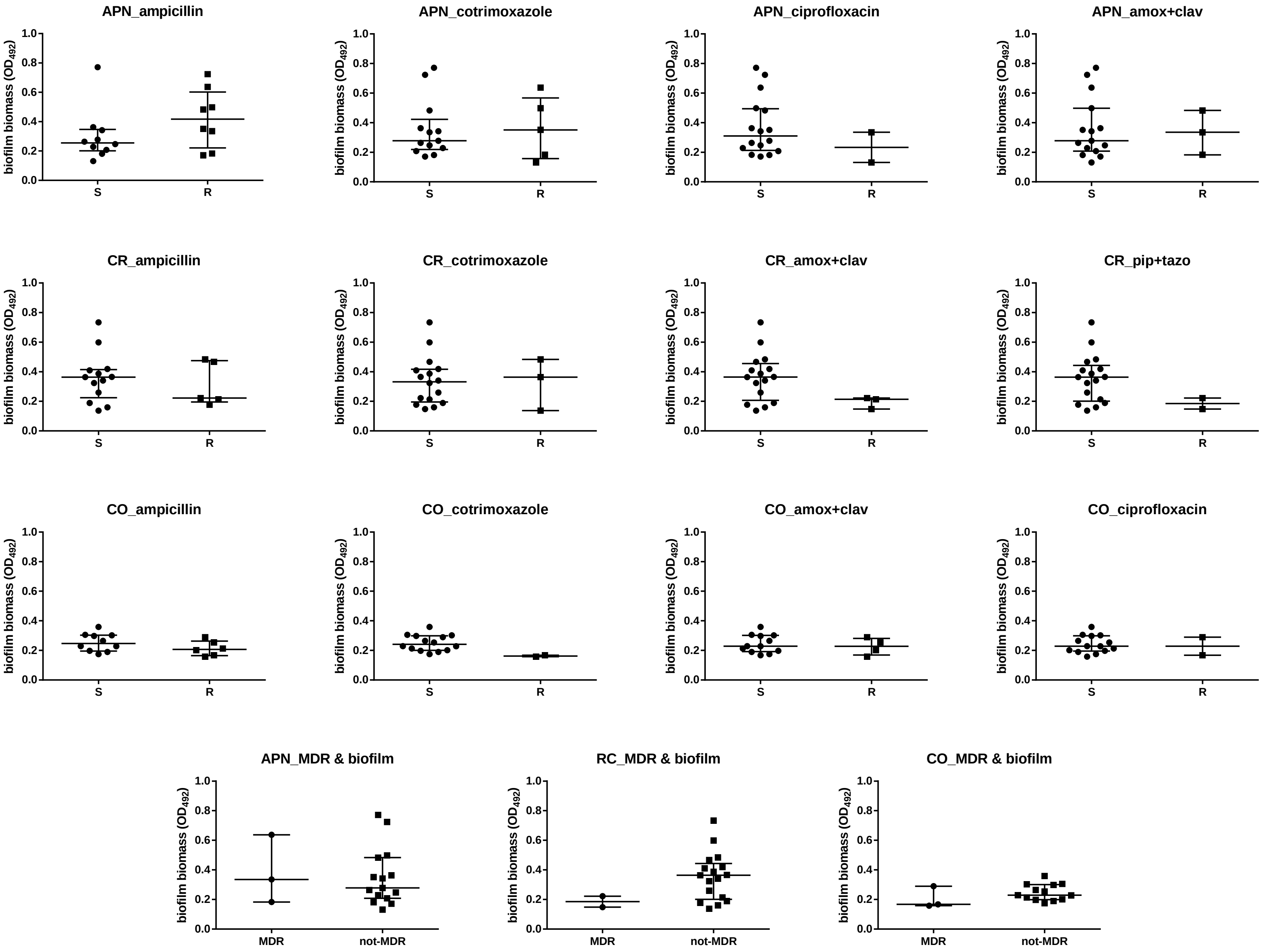
APN_ampicillin
APN_cotrimoxazole
APN_ciprofloxacin
APN_amox+clav
CR_ampicillin
CR_cotrimoxazole
CR_amox+clav
CR_pip+tazo
CO_ampicillin
CO_cotrimoxazole
CO_amox+clav
CO_ciprofloxacin
APN_MDR & biofilm
RC_MDR & biofilm
CO_MDR & biofilm
biofilm biomass (OD_{492})
0.0
0.2
0.4
0.6
0.8
1.0
S
R
MDR
not-MDR
